# Proteomics and metabonomics analyses of Covid-19 complications in patients with pulmonary fibrosis

**DOI:** 10.1038/s41598-021-94256-8

**Published:** 2021-07-16

**Authors:** Jianrong Yang, Chunxia Chen, Wan Chen, Luying Huang, Zhao Fu, Kun Ye, Liwen Lv, Zhihuang Nong, Xing Zhou, Wensheng Lu, Mei Zhong

**Affiliations:** 1 Hepatobiliary Surgery, the People’s Hospital of Guangxi Zhuang Autonomous Region, Nanning, 530021 Guangxi People’s Republic of China; 2 Department of Research Center of Medical Sciences, the People’s Hospital of Guangxi Zhuang Autonomous Region, Nanning, 530021 Guangxi People’s Republic of China; 3 Department of Pharmaceutical Sciences (Shenzhen), Sun Yat-Sen University, Guangzhou, 510006 Guangdong People’s Republic of China; 4 Department of Emergency, the People’s Hospital of Guangxi Zhuang Autonomous Region, Nanning, 530021 Guangxi People’s Republic of China; 5 Department of Respiratory Diseases, the People’s Hospital of Guangxi Zhuang Autonomous Region, Nanning, 530021 Guangxi People’s Republic of China; 6 Department of Radiology, the People’s Hospital of Guangxi Zhuang Autonomous Region, Nanning, 530021 Guangxi People’s Republic of China; 7 Department of Nephrology, the People’s Hospital of Guangxi Zhuang Autonomous Region, Nanning, 530021 Guangxi People’s Republic of China; 8 Department of Pharmacy, the People’s Hospital of Guangxi Zhuang Autonomous Region, Nanning, 530021 Guangxi People’s Republic of China; 9 Department of Endocrinology, the People’s Hospital of Guangxi Zhuang Autonomous Region, 6 Taoyuan Road, Nanning, 530021 Guangxi People’s Republic of China

**Keywords:** Cell biology, Mechanisms of disease, Molecular medicine

## Abstract

Pulmonary fibrosis is a devastating disease, and the pathogenesis of this disease is not completely clear. Here, the medical records of 85 Covid-19 cases were collected, among which fibrosis and progression of fibrosis were analyzed in detail. Next, data independent acquisition (DIA) quantification proteomics and untargeted metabolomics were used to screen disease-related signaling pathways through clustering and enrichment analysis of the differential expression of proteins and metabolites. The main imaging features were lesions located in the bilateral lower lobes and involvement in five lobes. The closed association pathways were FcγR-mediated phagocytosis, PPAR signaling, TRP-inflammatory pathways, and the urea cycle. Our results provide evidence for the detection of serum biomarkers and targeted therapy in patients with Covid-19.

## Introduction

In December 2019, pneumonia with an unknown cause became prevalent in Wuhan, China. When RNA was extracted and sequenced from these patients in samples of bronchoalveolar lavage fluid, the causative agent was found to be a novel betacoronavirus, SARS-CoV-2^1^. Subsequently, the outbreak was declared a public health emergency, and the novel coronavirus disease was named Covid-19 by the World Health Organization (WHO)^2^. As of November, 2020, over 42 million Covid-19 cases and 1 million deaths have been confirmed globally. However, the epidemic continues to spread, especially in the Americas and southeast Asia^3^. Currently, there are no effective therapies to treat Covid-19, although countries continue to develop new vaccines^4,5^. Therefore, developing new ways to control the progression of disease is urgent.

Pulmonary fibrosis is a phenomenon in patients with Covid-19, as evidenced by CT imaging and autopsy^6,7^. It is a progressive lung disease, and lung transplantation may be the only treatment to elevate life expectancy^8^. Recently, the Arg-Gly-Asp integrin-binging domain in SARS-CoV-2 spike protein was recognized by some researchers as one of the target pathways^9,10^, and the inflammatory factor IL-1 was implicated as possibly mediating its binding integrins^11^. TGF-β orchestrates multifunctional aspects of fibrogenesis, such as proliferation, deposition of extracellular matrix and fiber formation^12,13^. The TGF-β pathway is the main antifibrotic therapeutic pathway^14,15^. However, more pathogenetic profibrotic pathways need to be explored.

Proteomics and metabonomics are useful methods to screen markers of disease occurrence through differential expression of proteins and metabolic compounds^16,17^. In this study, we combined proteomic and metabonomic data with chest CT imaging to investigate the mechanism of pulmonary fibrosis. For the first time, to study the related pathways of fibrosis progression in Covid-19, patients with pulmonary fibrosis were divided into a nonprogressive group and a progressive group according to the degree of fibrosis progression. Our research may provide useful clues and strategies for controlling the epidemic of Covid-19 in other countries worldwide, particularly the prognosis and treatment of pulmonary fibrosis.

## Results

### Patient demographics and laboratory findings

By 17 Mar 2020, the clinical data of 85 laboratory-confirmed SARS-CoV-2-infected cases were collected in Guangxi, China. The median age for all patients was 41 years, with an interquartile range of 29–50.5 years (Tables 1 and S1). Sixty percent of the patients were female.

**Table 1:** Baseline characteristics of patients with Covid-19.

Characteristics	All patients (n = 85)	Nonpulmonary fibrosis (n = 41)	Pulmonary fibrosis (n = 44)
Age, median (interquartile), years	41 (29–50.5)	31 (17.5–42)	48 (37.5–62.8)
**Sex**			
Male	34 (40.0)	18 (43.9)	16 (36.4)
Female	51 (60.0)	23 (56.1)	28 (63.6)
**WBCs (× 10**^**9**^**/L; normal range 3.6–10)**	5.9 (4.5–6.9)	6.0 (5.1–7.8)	6.1 (4.7–7.6)
Increased	9 (10.6)	4 (9.8)	5 (11.4)
Decreased	6 (7.0)	2 (2.5)	4 (9.1)
**Neutrophils (× 10**^**9**^/**L; normal range 1.7–7)**	3.2 (2.4–4.5)	2.9 (2.3–4.1)	3.8 (2.5–5.0)
Increased	6 (7.1)	1 (2.4)	5 (11.4)
Decreased	7 (8.2)	4 (9.8)	3 (6.8)
**Lymphocytes (× 10**^**9**^/**L; normal range 0.7–4)**	1.9 (1.4–2.4)	2.1 (1.8–2.7)	1.6 (1.1–2.1)
Increased	5 (5.9)	5 (12.2)	0 (0)
Decreased	5 (5.9)	1 (2.4)	4 (9.1)
Erythrocyte sedimentation rate, median (interquartile), mm/h	34 (18.0–56)	24 (9.8–45.3)	41.5 (28.0–65.0)
C-reactive protein ≥ 10 mg/L	20 (23.5)	7 (17.1)	13 (29.5)
Hypersensitive C-reactive protein ≥ 10 mg/L	21 (24.7)	8 (19.5)	13 (29.5)
Procalcitonin ≥ 0.05 ng/mL	24 (28.2)	6 (14.6)	18 (40.9)
D-dimer > 0.5 mg/L	50 (58.8)	22 (53.7)	28 (63.6)
Oxygenation index > 300	73 (85.9)	36 (87.8)	37 (84.1)

Except where indicated, data are cases of patients, with percentages in parentheses.

On admission, white blood cells (WBCs) were below the normal range in 9 (10.6%) patients and above the normal range in 6 (7.0%) patients. Seven (8.2%) patients showed neutropenia (neutrophil count < 1.7 × 10^9^), and 5 (5.9%) showed lymphopenia (lymphocyte count < 0.7 × 10^9^). The erythrocyte sedimentation rate (ESR) was higher in pulmonary fibrosis patients (median level 41.5 mm/h [interquartile 28.0–65.0]) than in nonpulmonary fibrosis patients (median level 24 mm/h [interquartile 9.8–45.3]). Levels of C-reactive protein, hypersensitive C-reactive protein, procalcitonin, D-dimer, and oxygenation index were increased in 13 (29.5%), 13 (29.5%), 18 (40.9%), 28 (63.6%) and 37 (84.1%) pulmonary fibrosis patients, respectively, while they were increased in 7 (17.1%), 8 (19.5%), 6 (14.6%), 22 (53.7%) and 36 (87.8%) nonpulmonary fibrosis patients.

### Chest CT findings

**Figure 1:**
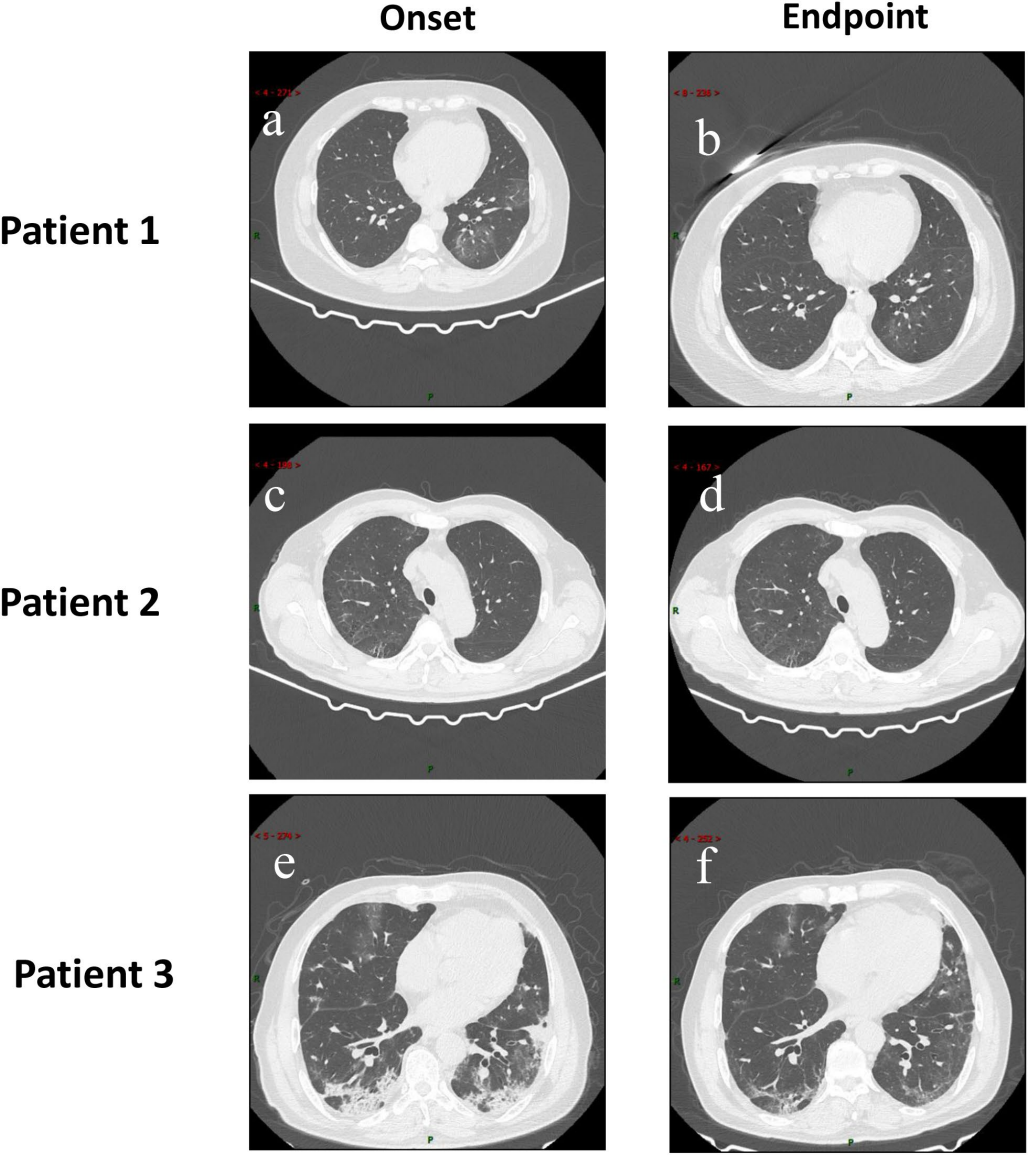
Representative chest CT images. (**a**) Ground-glass opacities in the dorsal segment of the left lower lobe of lung were seen in a 44-year-old female on admission, and partial absorption of lung lesions before discharge (**b**), Multiple fibrotic stripes shadows in dorsal segment of the lower lobe of the right lung were seen in a 62-year-old male after symptom onset (**c**), however, no obvious changes were observed before discharge (**d**, **e**), multiple fibrotic stripes shadows and patchy dense shadow (exudative lesion) were shown in a 65-year-old severe male on admission. (**f**) The exudative lesions were less and the fibrosis lesions were more than before in 20 days after hospitalization.

Of the 85 patients, 11 (12.9%) had no abnormality at admission on chest CT, including 10 (24.4%) of 41 nonpulmonary fibrosis patients and 1 (2.3%) of 44 pulmonary fibrosis patients (Table 2 and Fig. 1). Most of the patients (68 [80%]) had the single form of ground-glass opacities in the process of disease, while a few patients (10 [11.8%]) had the mixed form of ground-glass opacities and consolidation. Thirty-eight (44.7%) patients had fibrosis on admission, and 2 had fibrosis before discharge. In the nonpulmonary fibrosis group, the numbers of involved lobes were usually 1, 2, and 5. In contrast, the number of involved lobes in the pulmonary fibrosis group was mainly 5. The total lung severity score of the pulmonary fibrosis group was higher than that of the nonpulmonary fibrosis group at both stages of admission and discharge. Lesions were mainly peripheral and central distribution in the pulmonary fibrosis group (20 [50.0%]) at admission and then turned to peripheral distribution (27 [61.4%]) before discharge, whereas lesions were mainly peripherally distributed in the nonpulmonary fibrosis group. Importantly, almost half of the pulmonary fibrosis patients exhibited linear consolidation, bronchiectasis, and pleural incrassation in both stages of admission and discharge.

**Table 2:** Chest CT findings of patients with Covid-19.

Finding	All patients		Nonpulmonary fibrosis		Pulmonary fibrosis	
	Onset (n = 85)	Endpoint (n = 71)	Onset (n = 41)	Endpoint (n = 31)	Onset (n = 44)	Endpoint (n = 40)
No abnormality	11 (12.9)	4 (5.6)	10 (24.4)	4 (12.9)	1 (2.3)	0 (0)
**Lesion morphology**						
Ground-glass opacities	68 (80)	59 (83.1)	26 (63.4)	20 (64.5)	42 (95.5)	39 (97.5)
Ground-glass opacities with consolidation	10 (11.8)	3 (4.2)	6 (2.4)	0 (0)	4 (9.1)	3 (7.5)
Fibrotic stripes	38 (44.7)	40 (56.3)	0 (0)	0 (0)	38 (86.4)	40 (100)
**NO. of lobes affected**						
0	11 (12.9)	4 (5.6)	10 (24.4)	4 (12.9)	1 (2.3)	0 (0)
1	15 (17.6)	12 (16.9)	12 (29.3)	9 (29.0)	3 (6.8)	3 (7.5)
2	7 (8.2)	8 (11.3)	6 (14.6)	5 (16.1)	1 (2.3)	3 (7.5)
3	10 (11.8)	5 (7.0)	4 (9.8)	2 (6.5)	6 (13.6)	3 (7.5)
4	3 (3.5)	0 (0)	1 (2.4)	0 (0)	2 (4.5)	0 (0)
5	38 (44.7)	38 (53.5)	7 (17.1)	7 (22.6)	31 (70.5)	31 (77.5)
Total lung severity score, median (interquartile)	4 (1.5–8.0)	5 (2.0–9.0)	2 (0.5–3.0)	2 (1.0–5.0)	7 (5.0–10.0)	6 (5.0–11.5)
**Distribution of lesions**						
Peripheral	33 (38.8)	31 (43.7)	17 (41.5)	11 (35.5)	16 (36.4)	20 (50.0)
Peripheral and central	34 (40.0)	24 (33.8)	7 (17.1)	7 (22.6)	27 (61.4)	17 (42.5)
**Other findings**						
Linear	50 (58.8)	44 (62.0)	11 (26.8)	9 (29.0)	39 (88.6)	35 (87.5)
Consolidation	27 (31.8)	18 (25.4)	1 (2.4)	1 (3.2)	26 (59.1)	17 (42.5)
Discrete pulmonary nodules	15 (17.6)	8 (11.3)	9 (22.0)	5 (16.1)	6 (13.6)	3 (7.5)
Pulmonary emphysema	14 (16.5)	11 (15.5)	6 (14.6)	5 (16.1)	8 (18.2)	6 (15.0)
Bronchiectasis	29 (34.1)	27 (38.0)	3 (7.3)	5 (16.1)	26 (59.1)	22 (55.0)
Pleural effusion	4 (4.7)	2 (2.8)	0 (0)	1 (3.2)	4 (9.1)	1 (2.5)
Pleural incrassation	43 (50.6)	37 (52.1)	6 (14.6)	5 (16.1)	37 (84.1)	32 (80)

Except where indicated, data are cases of patients, with percentages in parentheses.

Then, we further evaluated the distribution of fibrosis in Covid-19 patients complicated with pulmonary fibrosis, including nonprogressive pulmonary fibrosis patients and progressive pulmonary fibrosis patients (Table 3, Fig. 1c–f.). In the progressive pulmonary fibrosis group, on admission, 6 (20%) patients had no fibrosis, and 14 (46.7%) patients had fibrosis in 5 lobes. However, 23 (76.7%) patients had fibrosis involving 5 lobes before discharge. Pulmonary fibrosis was widely distributed in both groups, especially in the left lower lobe and the right lower lobe.

**Table 3:** Chest CT evaluations of Covid-19 complicated with pulmonary fibrosis patients.

Finding	All pulmonary fibrosis patients (n = 40)		Nonprogressive pulmonary fibrosis (n = 10)		Progressive pulmonary fibrosis (n = 30)	
	Onset	Endpoint	Onset	Endpoint	Onset	Endpoint
**NO. of fibrotic lobes**						
0	6 (15.0)	0 (0)	0 (0)	0 (0)	6 (20.0)	0 (0)
1	3 (7.5)	2 (5.0)	2 (20.0)	2 (20.0)	1 (3.3)	0 (0)
2	1 (2.5)	1 (2.5)	0 (0)	0 (0)	1 (3.3)	1 (3.3)
3	6 (15.0)	4 (10.0)	2 (20.0)	2 (20.0)	4 (13.3)	2 (6.7)
4	5 (12.5)	7 (17.5)	1 (10.0)	3 (30.0)	4 (13.3)	4 (13.3)
5	19 (47.5)	26 (65.0)	5 (50.0)	3 (30.0)	14 (46.7)	23 (76.7)
**Frequency of fibrotic lobe involvement**						
Right upper lobe	22 (55.0)	31 (77.5)	6 (60.0)	5 (50.0)	16 (53.3)	26 (86.7)
Right middle lobe	28 (70.0)	34 (85.0)	7 (70.0)	7 (70.0)	21 (70.0)	27 (90.0)
Right lower lobe	32 (80.0)	36 (90.0)	9 (90.0)	7 (70.0)	23 (76.7)	29 (96.7)
Left upper lobe	27 (67.5)	34 (85.0)	6 (60.0)	7 (70.0)	21 (70.0)	27 (90.0)
Left lower lobe	31 (77.5)	39 (97.5)	9 (90.0)	9 (90.0)	22 (73.3)	30 (100.0)
*Total pulmonary fibrosis severity score, median (interquartile)	5.0 (2.3–7.0)	7.0 (5.0–11.5)	5.0 (3.5–5.3)	4.5 (3.5–5.3)	6.0 (1.8–7)	8.0 (5.0–14.0)

Except where indicated, data are cases of patients, with percentages in parentheses. *The corresponding score of each lobe in the five lobes was 0, 1, 2, 3, 4, respectively, and the corresponding affected area was 0, 1–25%, 26–50%, 51–75%, 76–100%, which was referred to the total lung severity score.

### Proteomic and metabolomic analysis of Covid-19 patients with pulmonary fibrosis

**Figure 2:**
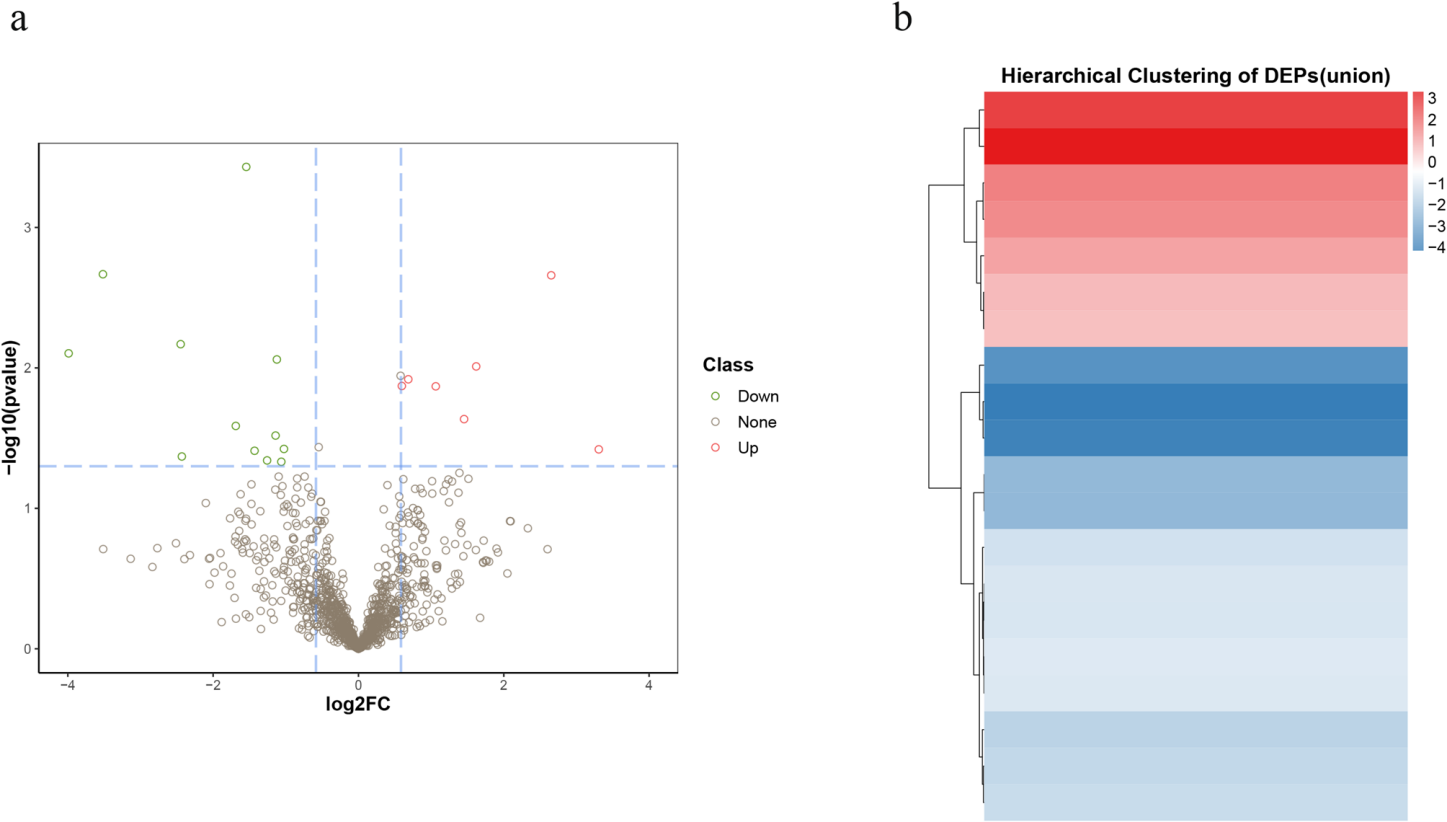
Differentially expressed proteins (DEPs) were identified and clustered in Covid-19 patients with pulmonary fibrosis. (**a**) Volcano plots of total protein levels. Red dots, significantly upregulated proteins; green dots, significantly downregulated proteins. (**b**) Cluster chart of DEPs. Red rows, significantly upregulated proteins; blue rows present significantly downregulated proteins.

The Q-Exactive HF (Thermo Fisher Scientific, San Jose, CA) instrument collected mass spectrometry data from 27 samples (randomly sampled from each group) in DIA mode (Table S2). A total of 8055 peptide fragments and 1079 proteins were quantified (Table S3). A heat map of the sample correlation analysis is shown in Fig. S1a. After calculating the fold difference between Covid-19 patients with pulmonary fibrosis (n = 21) and Covid-19 patients without fibrosis (n = 6) by MSstats software, 20 differentially expressed proteins were found, including 13 downregulated and 7 upregulated proteins (Fig. 2a,b). GO and KEGG^18^ enrichment analysis indicated that DEPs were related to the immune system, biological adhesion, and glycosaminoglycan degradation (Fig. S1b–d and Table S4).

**Figure 3:**
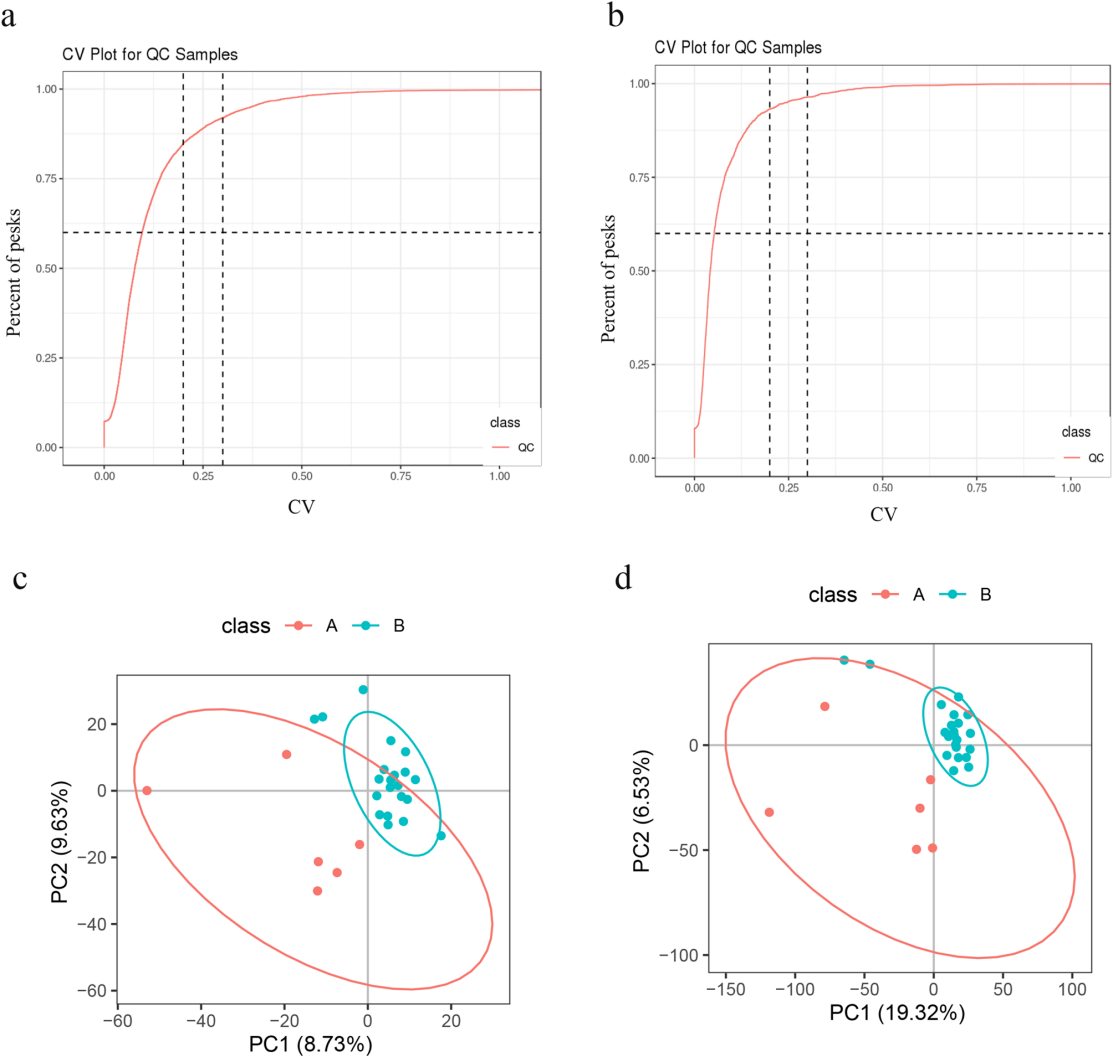
Quality control of samples in Covid-19 patients with pulmonary fibrosis. (**a**, **b**) Coefficient of variation (CV) plots for positive and negative ion modes. (**c**,**d**) PLS-DA model plots for positive and negative ion modes. A, Covid-19 patients without pulmonary fibrosis (n = 6). B, Covid-19 patients with pulmonary fibrosis (n = 22).

**Figure 4:**
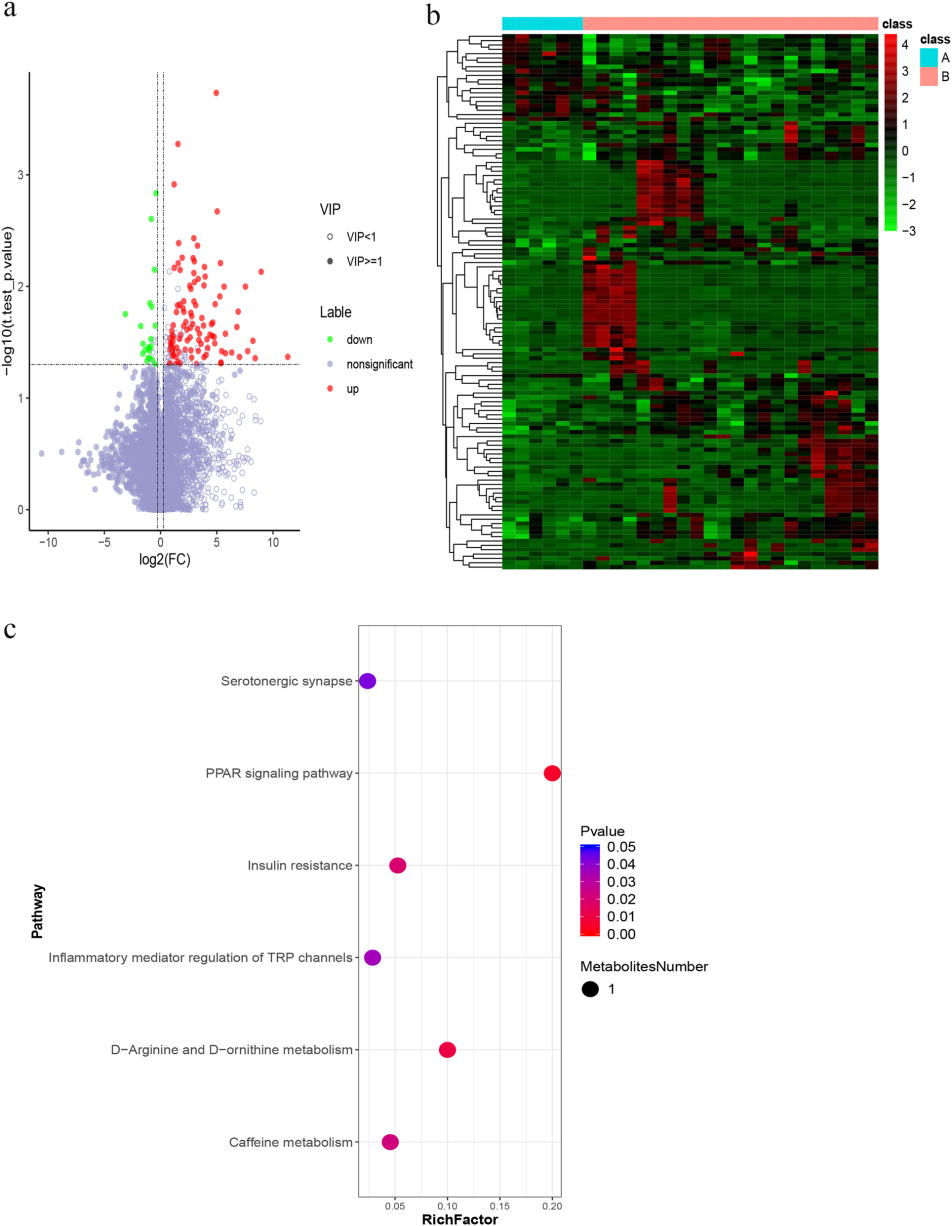
Positive differentially expressed metabolites were identified, clustered and enriched in Covid-19 patients with pulmonary fibrosis. (**a**) Volcano plots of positive compounds. Red dots, significantly upregulated metabolites; green dots, significantly downregulated metabolites. (**b**) Heatmap of different positive metabolites (“pheatmap” package in R software v3.5.0, https://www.r-project.org/). Each row denotes a different metabolite, and each column denotes a sample. A, Covid-19 patients without pulmonary fibrosis (n = 6). B, Covid-19 patients with pulmonary fibrosis (n = 22). (**c**) Bubble plot of KEGG enrichment of positive metabolic pathways. The size of the circle dot denotes the number of different metabolites. RichFactor is defined as the number of differential metabolites annotated to the pathway divided by all identified metabolites annotated to the pathway.

For metabolomic analysis, the data of positive and negative ions were collected from 28 samples (randomly sampled from each group) to improve the metabolite coverage. The coefficient of variation (CV) plot shows that there was a high degree of repeatability for samples (Fig. 3a,b). PLS-DA (Fig. 3c,d) was used to establish the relationship model between metabolite expression and sample types to predict sample categories and to determine the differential metabolism among groups. A total of 3020 positive compounds with identification information (Table S5) and 978 negative compounds were identified (Table S6), among which 102 positive compounds were upregulated and 21 were downregulated (Fig. 4a), while 38 negative compounds were upregulated and 3 were downregulated (Fig. S2a) between Covid-19 patients with pulmonary fibrosis (n = 22) and Covid-19 patients without fibrosis (n = 6). The cluster analysis of different metabolites is shown in Fig. 4b and Fig. S2b. Based on the KEGG database, pathways including the peroxisome proliferator-activated receptor (PPAR) signaling pathway, D-arginine and D-ornithine metabolism, inflammatory mediator regulation of TRP channels and alpha-linolenic acid metabolism were significantly enriched (Fig. 4c, S2c and Table S7).

### Proteomic and metabolomic analyses of Covid-19 patients with progressive pulmonary fibrosis

**Figure 5:**
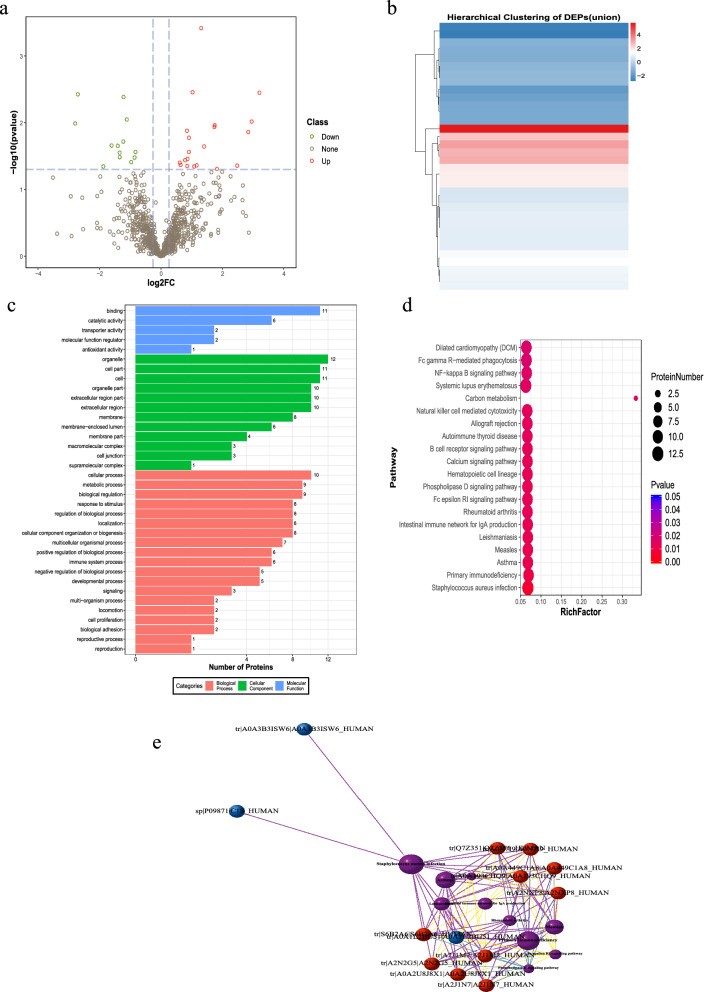
Differentially expressed proteins (DEPs) were identified and analyzed using proteomics in Covid-19 patients with progressive pulmonary fibrosis. (**a**) Volcano plots of total protein level. (**b**) Cluster chart of DEPs. (**c**) Enrichment analysis of DEPs by GO function. (**d**) Bubble plot of the top 20 KEGG enrichment pathways of DEPs. RichFactor is defined as the number of differential metabolites annotated to the pathways divided by all identified metabolites annotated to the pathway. (**e**) Pathway network of DEPs (“igraph” package in R software v3.5.0, https://www.r-project.org/). The red and blue dots represent up-regulated and down-regulated differential proteins, respectively. The purple balls represent the top 10 pathways of enrichment. The area is directly proportional to the enrichment degree.

To further search for factors related to the progression of pulmonary fibrosis, we divided the patients with pulmonary fibrosis into a progressive group and a nonprogressive group and continued to conduct proteomic and metabolomic tests on their serum. It was found that 34 proteins were differentially expressed between the progressive pulmonary fibrosis Covid-19 patients (n = 16) and the nonprogressive pulmonary fibrosis Covid-19 patients (n = 5) (Fig. 5a). After clustering (Fig. 5b) and enrichment analysis of DEPs (Fig. 5c–e, and Table S4), it was found that DEPs were mainly distributed in the immune system, including primary immunodeficiency, intestinal immune network for IgA production, FcγR-mediated phagocytosis, natural killer cell-mediated cytotoxicity, the B cell receptor signaling pathway, and the FcεRI signaling pathway. Moreover, the NF-kappa B, calcium, hematopoietic cell lineage, and phospholipase D signaling pathways were also involved.

**Figure 6:**
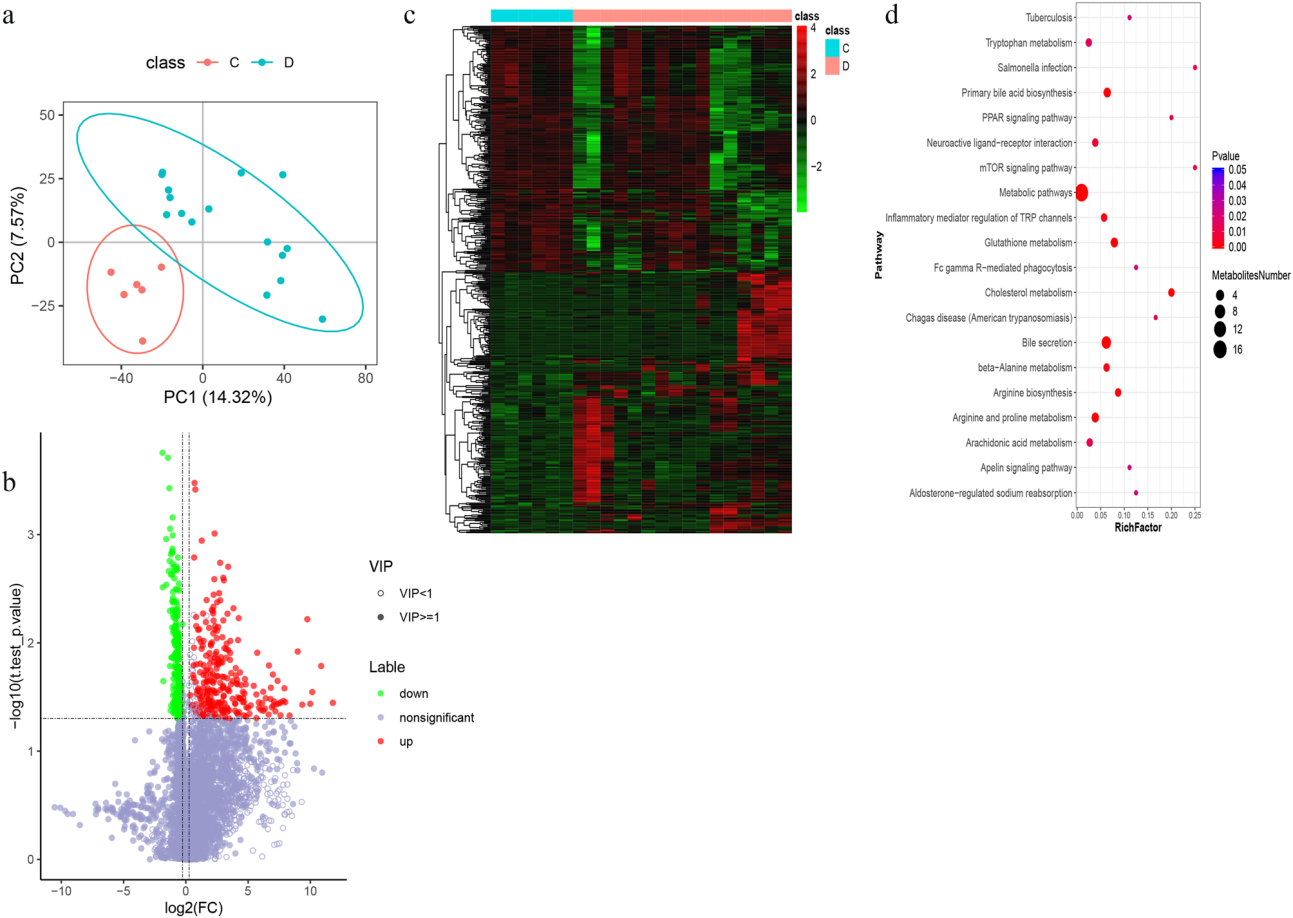
Positive differentially expressed metabolites were identified and analyzed using proteomics in Covid-19 patients with progressive pulmonary fibrosis. (**a**) PLS-DA model plot for positive ion mode. (**b**) Volcano plots of positive compounds. (**c**) Heatmap of different positive metabolites (“pheatmap” package in R software v3.5.0, https://www.r-project.org/). (**d**) Bubble plot of the top 20 KEGG enrichment of positive metabolic pathways. C, Nonprogressive pulmonary fibrosis of Covid-19 patients. D, Progressive pulmonary fibrosis of Covid-19 patients. RichFactor is defined as the number of differential metabolites annotated to the pathway divided by all identified metabolites annotated to the pathway.

In terms of the metabolomic analysis, we found that the PLS-DA model had a good prediction effect (Figs. 6a and S3a). A total of 511 differential positive metabolites and 160 negative metabolites were screened (Figs. 6b and S3b) between the progressive pulmonary fibrosis Covid-19 patients (n = 16) and the nonprogressive pulmonary fibrosis Covid-19 patients (n = 6) and clustered (Figs. 6c and S3c). Using the KEGG database, metabolic pathway enrichment analysis revealed the following pathways with differential metabolite enrichments: PPAR signaling pathway, mTOR signaling pathway, inflammatory mediator regulation of TRP channels, FcγR-mediated phagocytosis, arginine metabolism, and alpha-Linolenic acid metabolism (Figs. 6d, S3d and Table S7).

### Correlation analysis between proteomics and metabolomics in Covid-19 patients complicated with progressive pulmonary fibrosis

**Figure 7:**
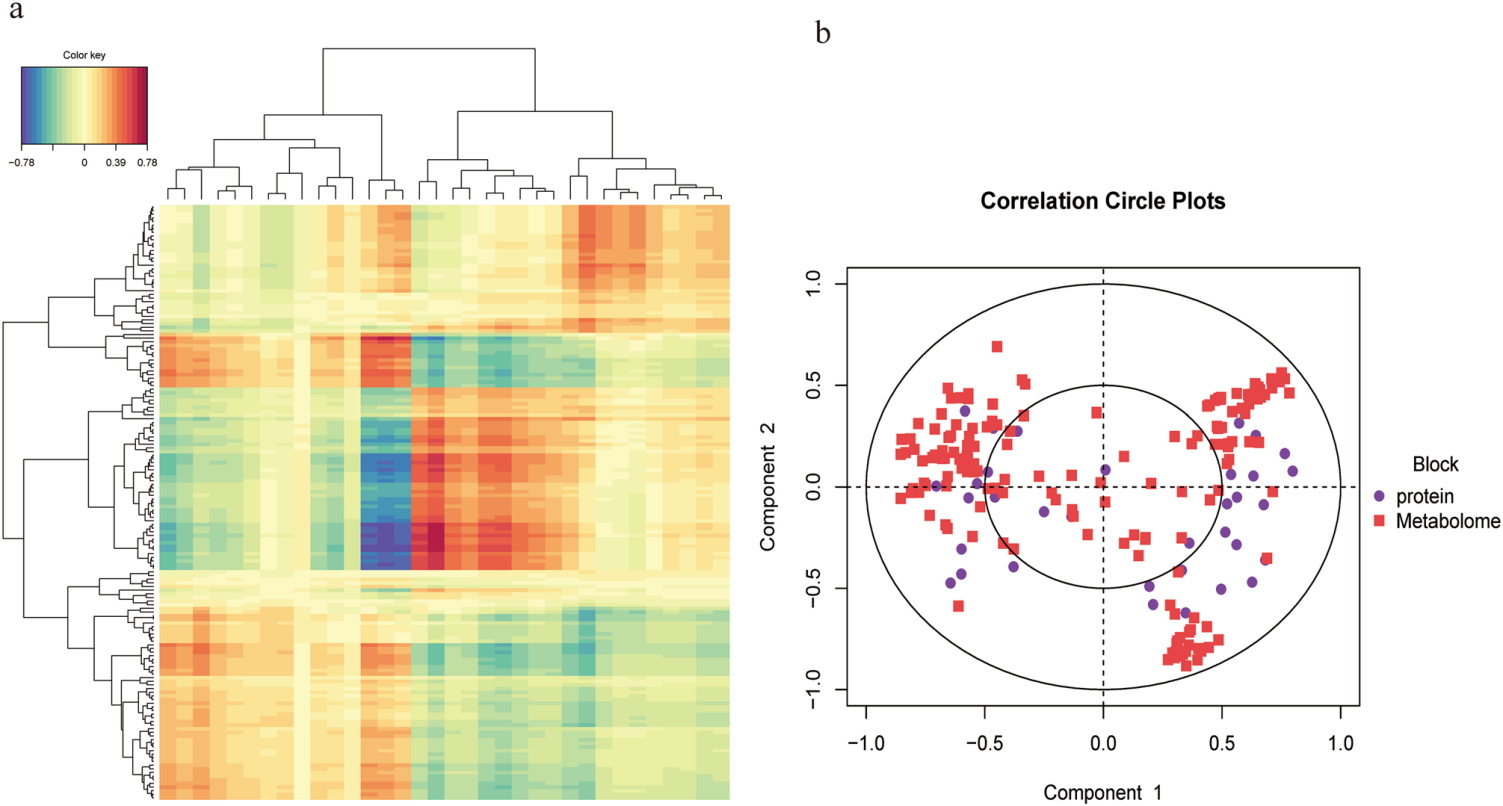
Association analysis of proteomics and metabolomics in Covid-19 patients with progressive pulmonary fibrosis. (**a**) Cluster heat map of the correlation between proteomics and metabolomics (“pheatmap” package in R software v3.5.0, https://www.r-project.org/). Each row denotes a differentially expressed metabolite, and each column denote a DEP. Blue, negative correlation; Red, positive correlation. (**b**) Concentric circles of correlation between differential proteins and differential metabolites. If the angle between a differential protein and differential metabolite is an acute angle (less than 90 degrees), the correlation is positive. If the angle between the differential protein and the differential metabolite is an oblique angle (greater than 90 degrees and less than 180 degrees), the correlation is negative. Starting from the center of the circle, the lines are connected to the differential metabolites and differential proteins. The longer the connection length, the stronger the relationship.

To further explore the relationship between biological network regulation, we conducted proteome and metabolomic association analyses between the nonprogressive pulmonary fibrosis group and the progressive pulmonary fibrosis group. By performing regularized canonical correlation analysis, correlation clustering of DEPs and differential metabolites can be observed in the heatmap (Fig. 7a). After multivariate dimensionality reduction and relationship visualization of omics (Fig. 7b), it was found that the DEPs and differential metabolites in pulmonary fibrosis group (n = 21) were correlated with each other.

## Discussion

To our knowledge, this is the first paper describing an integrated multiomics study in Covid-19 complicated with pulmonary fibrosis patients at the molecular and ionic levels. First, after reviewing the medical records of 85 Covid-19 patients hospitalized in the People's Hospital of Guangxi Zhuang Autonomous Region Yongwu branch, we found that as many as 44.7% of the patients had fibrotic stripe shadows in the early onset, and the incidence rate was close to that of SARS outbreaks in 2003^19^. The most common manifestations of chest CT in Covid-19 fibrosis in our 85 cases were summarized as follows: lesions located in bilateral lower lobes, involved in five lobes, and complicated with linear consolidation, bronchiectasis, and pleural incrassation, which partially agreed with a previous report^6^. Second, we described the detailed profile of CT images in Covid-19 complicated with pulmonary fibrosis patients, especially focusing on progressive pulmonary fibrosis patients. Third, using proteomics and metabonomics methods, we explored the mechanism of the information pathway related to the occurrence and development of progressive fibrosis, which is expected to predict disease progression and to provide evidence for targeted therapy.

In recent years, proteomics has become an accurate method to screen disease biomarkers^20^ and has also been used in the pathogenesis of SARS-CoV-2 infection^16,17^. In this study, we found significant differential expression levels of immune system and biological adhesion markers and glycosaminoglycan degradation in pulmonary fibrosis patients. Previous experimental and clinical data demonstrated that the accumulation and deposition of glycosaminoglycan in the extracellular matrix contributed to progressive pulmonary fibrosis^21,22^. Moreover, glycosaminoglycan hyaluronan (HA) on AEC2s (stem cells in the adult lung) is critical for restoring renewal capacity and limiting the scope of fibrosis^23^. The general properties of cell adhesion molecules (CAMs) involve cell recruitment; they participate in cell–cell interactions and cell-extracellular matrix interactions^24^ and then promote the formation of fibrosis by intervention in the microenvironment^25^. For example, platelet/endothelial cell adhesion molecule-1 (PECAM-1) is expressed at intercellular junctions on platelets and leukocytes, including NK cells, T cells, monocytes and neutrophils^26^, which are involved in chemokine-mediated migration to the inflammatory site^27^. Moreover, PECAM-1 participates in the maintenance and restoration of vascular integrity following alveolar-capillary barrier disruption in ARDS^28^. In addition, CAMs modulate signal transduction by interacting with extracellular matrix proteins^29^. However, the results of this omics analysis are limited. The specific adhesion molecule biomarkers and the occurrence of diseases need further accurate detection.

It has been well documented that the immune system plays an important role in the occurrence and development of fibrosis^30,31^ and Covid-19^32^. The association of FCGR3B (encodes the lgG receptor FcγRIIIb) copy number variations with susceptibility to idiopathic pulmonary fibrosis (IPF) has been previously reported^33^. FcγR binds to serum amyloid P (SAP), inhibits the differentiation of monocytes into fibrocytes^30,31^ and affects neutrophil adhesion^30^. Moreover, SAP binds to Ca^2+^-dependent ligands to induce FcγR-mediated phagocytosis in vivo^34^. Recently, it was reported that segregated-nucleus-containing atypical monocytes, which were derived from FcεRI^+^ granulocyte/macrophage progenitors, were involved in fibrosis^35^. NK cells lacking FcεRIγ are associated with lower levels of fibrosis^36^. Furthermore, samples from 18 patients with IPF showed that the proportion of Breg cells (i.e., Breg cells to total B cells) was decreased, even though this proportion was positively correlated with the diffusion capacity of the lungs^37^. Consistent with the results of the above references, our study supported the cross-regulation among pathways of the immune system, calcium signaling and cell adhesion.

As a member of the nuclear receptor and transcription factor family, PPAR is a classical receptor for diabetic therapy^38^. However, recent emerging literature suggests a correlation between PPAR and lung fibrosis^39–41^. Activation of the PPARγ pathway provided protection against organ fibrosis in the lung. PPAR agonists are beneficial in treating preclinical models such as bleomycin-induced pulmonary fibrosis^42^ and genetically engineered mice^43^. Moreover, inhibiting PPARγ promoted fibroproliferative ARDS, which was observed in clinical bronchoalveolar lavage fluid (BAL) samples^44^. Our results from both pulmonary fibrosis patients and progressive pulmonary fibrosis patients in metabolomic analysis confirmed the differential expression of the PPAR signaling pathway, which suggested that PPAR signaling is a core pathway in the formation and development of lung fibrosis. TRP channels respond to a series of heterogeneous stimuli and are essential for pathophysiological homeostasis^45^. It has been reported that TRP channels participate in lung disease mainly through calcium signaling, recruitment of proinflammatory cells, and neurogenic inflammatory pathways^46^. In our study, pathway enrichment revealed that inflammatory mediator regulation of the TRP channel was an important pathway to regulate the occurrence and development of pulmonary fibrosis. Both arginine and ornithine are essential components in the urea cycle. L-arginine is the precursor of nitric oxide^47^, which is involved in the vascular system^48^. Treatment with L-arginine protects against pulmonary fibrosis progression in a mouse model^49^. D-arginine is an inactive form of L-arginine. The results from our study indicated that lung fibrosis was associated with dysfunction of the urea cycle metabolic pathway. D-arginine and D-ornithine could be used as an early warning and a sign of progression of pulmonary fibrosis disease.

This study has several limitations. First, although the 85 Covid-19 cases were all confirmed patients in our hospital, their numbers are relatively few and cannot represent the whole of China or the international situation or even secondary infection. Second, the CV of proteomics was large because the samples were taken from patients' serum. Third, the immune system, PPAR, and TRP pathways screened by proteomics and metabonomics in this study need to be further verified in vivo and in vitro, while the pathways related to nervous system diseases need to be further explored, although it has been reported that Covid-19 patients have nervous system damage^50^. Fourth, long-term follow-up of the progression of fibrosis is required.

In conclusion, in this study, proteomics, metabonomics, and correlation analysis were used to screen the related pathways of fibrosis formation and progression in Covid-19 patients, and the results showed that pathways including the immune system (especially the action of FcγR-mediated phagocytosis), PPAR signaling, TRP-inflammatory pathways, and the urea cycle were closely related. Our data provide new ideas for the treatment of Covid-19 and fibrosis patients.

## Methods

### Patient involvement and data collection

For this retrospective, single-center study, we recruited patients from Jan 15 to Mar 17, 2020, at the branches of the People's Hospital of Guangxi Zhuang Autonomous Region (Yongwu Hospital), a designated hospital for patients infected with SARS-CoV-2 in Guangxi Province. According to the guidelines of the National Health Commission of China, a case of confirmed infection with SARS-CoV-2 was diagnosed^51^. Patients enrolled in this study were laboratory confirmed. The study was approved by the ethics committee of the People's Hospital of Guangxi Zhuang Autonomous Region, China (KY-LW-2020–22) and was conducted in accordance with the Declaration of Helsinki. Informed consent was obtained from all patients.

### Laboratory confirmation

Specimens including nasal and pharyngeal swabs were collected for SARS-CoV-2 detection by real-time polymerase chain reaction (RT-PCR). Briefly, after total RNA was extracted by nucleic acid extraction and separation reagent (Sansure Biotech Inc., Changsha, China), one-step RT-PCR (bioPerfetus Technologies Co., Taizhou, China and Shanghai BioGerm Medical Technology Co., Shanghai, China) was performed using the following conditions: 50 °C for 10 min and 95℃ for 5 min, 40 cycles at 95 °C for 10 s and 55 °C for 40 s. The viral nucleic acid testing for patients was performed in the clinical laboratory of the People's Hospital of Guangxi Zhuang Autonomous Region Yongwu branch. Laboratory findings, including hematological and serological parameters and serum biochemistry, were traced.

### Chest CT

Eighty-five patients were imaged with a 64-detector row SOMATOM go. Top (Siemens Healthineers, Germany). The patient was in the supine position, and the images were collected at the end of inspiration. Chest CT was assessed at two time points: on admission and before discharge (or death). All images were independently reviewed by two radiologists with more than 5 years of working experience, and the final results were determined by consensus the two doctors.

Each image was evaluated for the following features: lesion morphology, fibrosis, number of lobes affected, total lung severity score, distribution of lesions, and other findings, including linear consolidation, discrete pulmonary nodules, pulmonary emphysema, bronchiectasis, pleural effusion and pleural incrassation. The total lung severity score was obtained by adding the involvement of each lobe^52,53^. The corresponding scores of each lobe in the five lobes were 0, 1, 2, 3, and 4, and the corresponding affected areas were 0, 1–25%, 26–50%, 51–75%, and 76–100%.

According to whether there were fibrosis bands on chest CT, the patients were divided into nonpulmonary fibrosis and pulmonary fibrosis groups. The latter group includes the nonprogressive pulmonary fibrosis group and the progressive pulmonary fibrosis group, based on the increased severity of fibrotic pulmonary lobe involvement.

### Proteome analysis

Serum samples were placed at 56 °C (water bath) for 30 min to inactivate the SARS-CoV-2 virus and were prepared as previously described with some modifications^16^. One hundred microliters of serum sample was denatured in 900 μL of L3 buffer containing 7 M urea, 2 M thiourea and 20 mM Tris (pH 8–8.5). The serum protein was extracted by the two following reactions: dithiothreitol (10 mM) at 37 °C (water bath) for 30 min and iodoacetamide at room temperature for 30 min (dark condition). The protein was enriched by a C18 column (Waters, USA) at a flow rate of 3 mL/min, washed with 0.1% formic acid (FA) and 75% ACN, and then dried in 75% ACN. Isolated proteins were digested with 1:20 trypsin overnight at 37 °C.

Peptides were fractionated using an LC-20AB system (Shimadzu, Japan) for high-pH reversed-phase fractionation and separated on a Gemini C18 column (5 μm × 4.6 mm × 250 mm; Waters, USA) using a multistep gradient from 5 to 95% buffer B (95% ACN, pH 9.8) at a flow rate of 1 mL/min for 64 min. Eluting peptides were collected every min, which were combined with a chromatographic elution peak diagram. Then, all the fractions were combined into a total of 10 fractions, which were then frozen and dried.

The fractions were redissolved in buffer A (2% ACN and 0.1% FA) and separated on an UltiMate 3000 UHPLC (Thermo Fisher Scientific) equipped with a C18 column (150 μm i.d, 1.8-μm particles, 35 cm long; Waters, USA). Peptides were eluted by a gradient from 5 to 80% buffer B. For data-dependent acquisition (DDA) mode detection, peptides were ionized by a nanoESI source and were then introduced into a Q-Exactive HF tandem mass spectrometer (Thermo Fisher Scientific, San Jose, CA) at a spray voltage of 1.9 kV. The MS spectra (350–1500 m/z) were acquired at a resolution of 120,000, a maximum injection time (MIT) of 100 ms and an AGC target value of 3 × 10^6^. The MS^2^ spectra (350–1500 m/z) were acquired at a resolution of 120,000, an MIT of 100 ms and an AGC target value of 1 × 10^5^. The data were identified using the Andromeda integrated by MaxQuant, and then the spectrum library was established.

For data-independent acquisition (DIA) mode detection, the same nano-LC system and gradient was used as DDA analysis. Briefly, the MS spectra (400–1250 m/z) were acquired at a resolution of 120,000, an MIT of 50 ms and an AGC target value of 1 × 10^6^. The ion fragmentation mode was HCD, the MIT was selected as automatic mode, the fragment ions were detected in Orbitrap, and the resolution was set to 30,000. The fragmentation energy was distributed as follows: 22.5, 25, 27.5. The data were analyzed and controlled by the mProphet algorithm.

### Metabolome analysis

The metabolites of serum samples were extracted by internal labeling and with a mixture of methanol and ACN at a ratio of 2:1. Ten microliters of supernatant from each sample was mixed into QC quality control samples to evaluate the repeatability and stability of the LC–MS analysis process. A Waters 2D UPLC (Waters, USA) series Q Exactive HF high-resolution mass spectrometer (Thermo Fisher Scientific, USA) was used for the separation and detection of metabolites.

A 5-μL aliquot of sample solution was injected on a BEH C18 column (100 mm × 2.1 mm, 1.7 μm; Waters, USA). The column oven was maintained at 55 °C. The positive ion mode mobile phases were aqueous solution containing 0.1% formic acid (A solution) and 100% methanol containing 0.1% formic acid (B solution). The negative ion mode mobile phase consisted of aqueous solution containing 10 mM ammonium formate (C solution) and 95% methanol containing 10 mM ammonium formate (D solution). The following gradient was used for elution: 0–1 min, 2% B/D solution; 1–9 min, 2–98% B/D solution; 9–12 min, 98% B/D solution; 12–12.1 min, 98–2% B/D solution; 12.1–15 min, 2% B/D solution. The flow rate was 0.35 mL/min. The range of mass spectrometry scanning with mass nucleus ratio was 70–1050, the first-stage resolution was 120,000, the AGC was 3 × 10^6^, and the MIT was 100 ms. According to the parent ion strength, top3 was selected for fragmentation, and secondary information was collected. The secondary resolution was 30,000, the AGC was 1 × 10^5^, and the MIT was 50 ms. The stepped energy was set to 20, 40, and 60 eV. The ion source was set at the following conditions: sheath gas flow rate, 40; aux gas flow rate, 10; spray voltage positive ion mode, 3.8; spray voltage negative ion mode, 3.2; capillary temperature, 320 °C; aux gas heater temperature, 350 °C.

### Statistical analysis

Continuous variables are represented as medians with interquartile ranges, while categorical variables are expressed as counts (%). The statistical analyses were performed using SPSS 13.0.

DDA data were identified by MaxQuant 1.5.3.30 (Max Planck Institutes) software and the UniProtKB/Swiss-Prot Homo sapiens database. Identification information that met FDR ≤ 1% was used to build the final library. The parameters were selected as follows: enzyme: trypsin; minimal peptide length: 7; PSM-level FDR: 0.01; protein FDR: 0.01; fixed modifications: carbamidomethyl (C); variable modifications: oxidation (M), acetyl (protein N-term). DIA data were analyzed by Spectronaut (Biognosys) software, which used iRT peptide to correct the retention time. Then, based on the target-decoy model applicable to SWATH-MS, the false-positive control was completed at FDR 1% to obtain significant quantitative results. The R package MSstats (Bioconductor) screened differential proteins according to the fold change > 1.5 and *P* value < 0.05, while screened differential metabolites according to variable importance for the projection (VIP) ≥ 1, fold change ≥ 1.2 or ≤ 0.83, and *P* value < 0.05. Meanwhile, differential proteins and differential metabolites were enriched by network pathway analysis database: gene ontology (GO) (http://www.geneontology.org.) and the kyoto encyclopedia of genes and genomes (KEGG). The protein–protein interaction network of the difference proteins was analyzed by STRING database (http://string-db.org). DEPs and differential metabolites were calculated by regularized canonical correlation analysis and were visualized using the plotVar functions in the mixOmics package.

## Supplementary Information

Supplementary Information 1.

Supplementary Information 2.

Supplementary Information 3.

Supplementary Information 4.

Supplementary Information 5.

Supplementary Information 6.

Supplementary Information 7.

Supplementary Information 8.

Supplementary Information 9.

Supplementary Information 10.

Supplementary Information 11.

## Data Availability

The mass spectrometry proteomics data have been deposited to the ProteomeXchange Consortium (http://proteomecentral.proteomexchange.org) via the iProX partner repository^54^ with the dataset identifier PXD024311. All data are fully available without restriction.
