# Supplementary figures and images for: Proteomics and metabonomics analyses of Covid-19 complications in patients with pulmonary fibrosis
Source: Sci Rep. 2021 Jul 16;11:14601. doi: 10.1038/s41598-021-94256-8 (PMC8285535; doi:10.1038/s41598-021-94256-8)

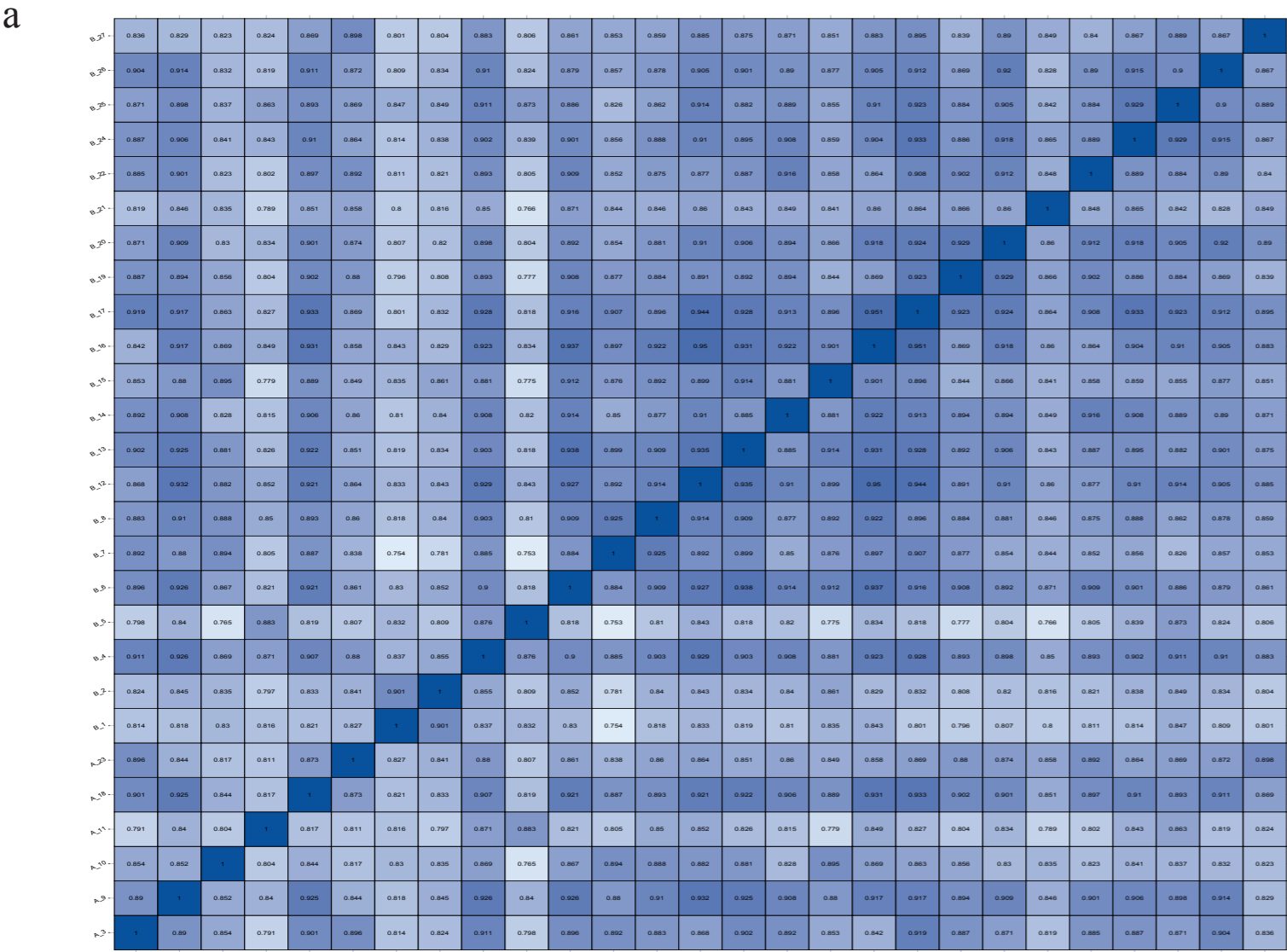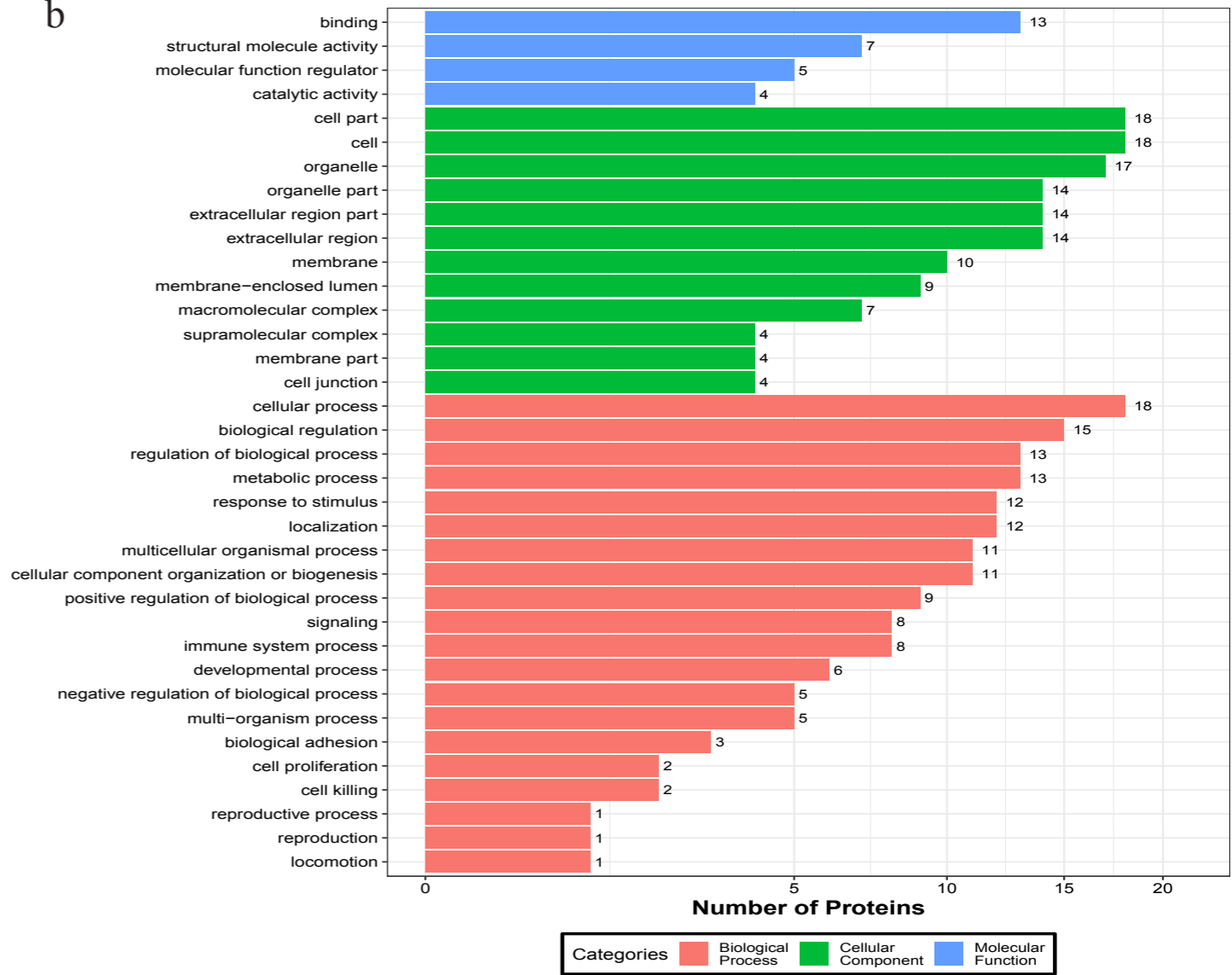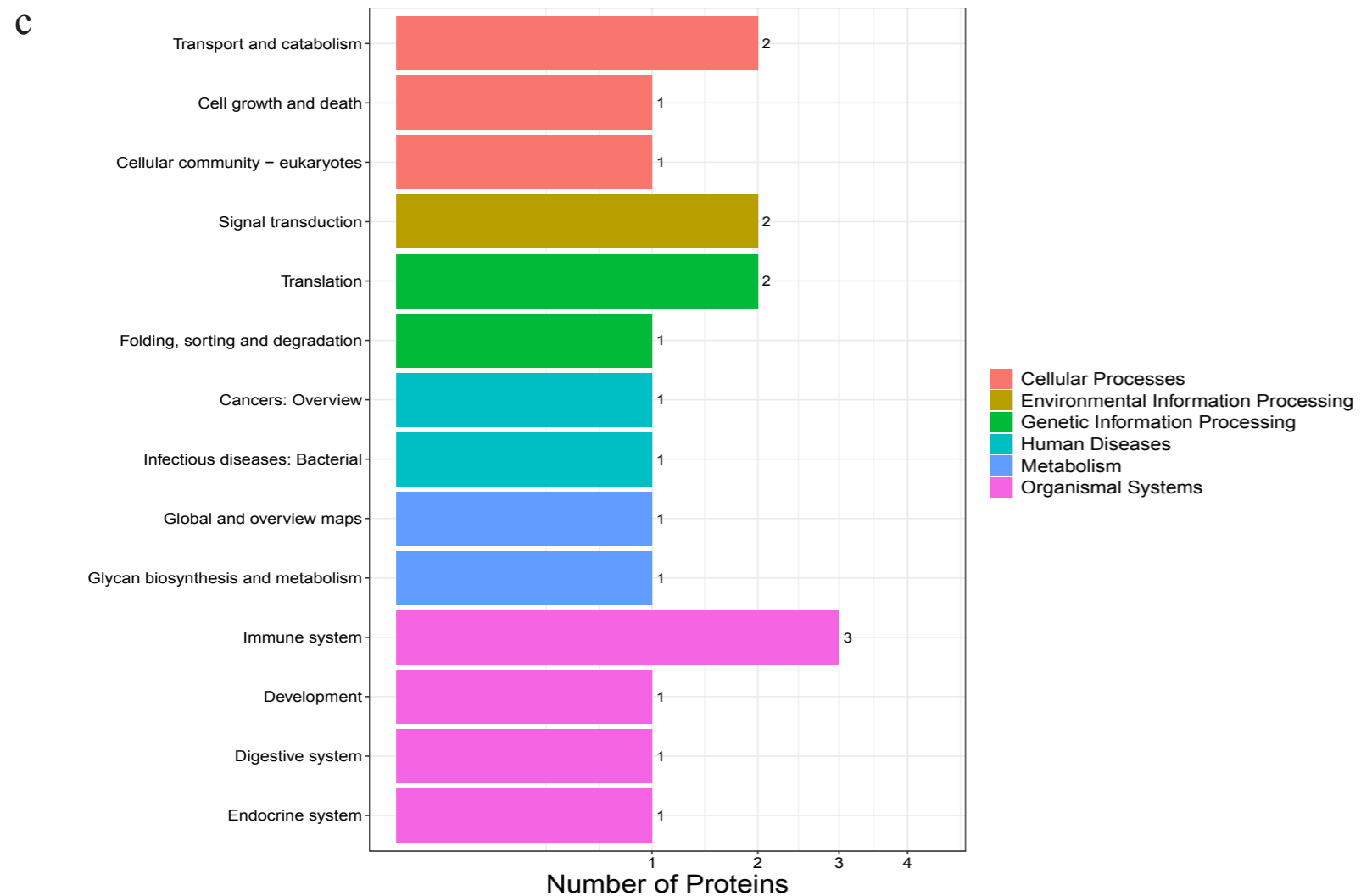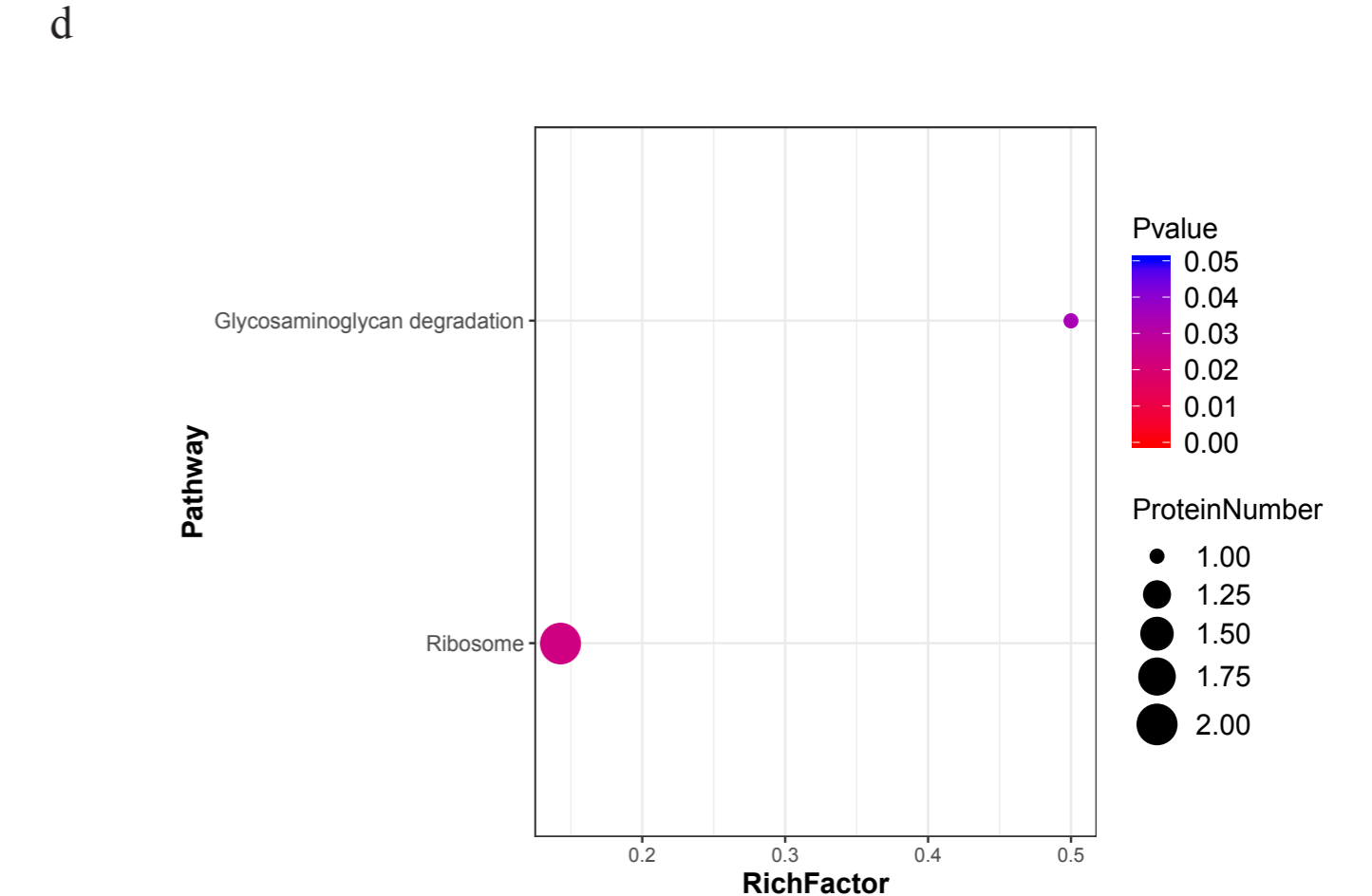

Supplement: Supplementary file 1 — Supplementary Information 1. [file 41598_2021_94256_MOESM1_ESM.pdf]

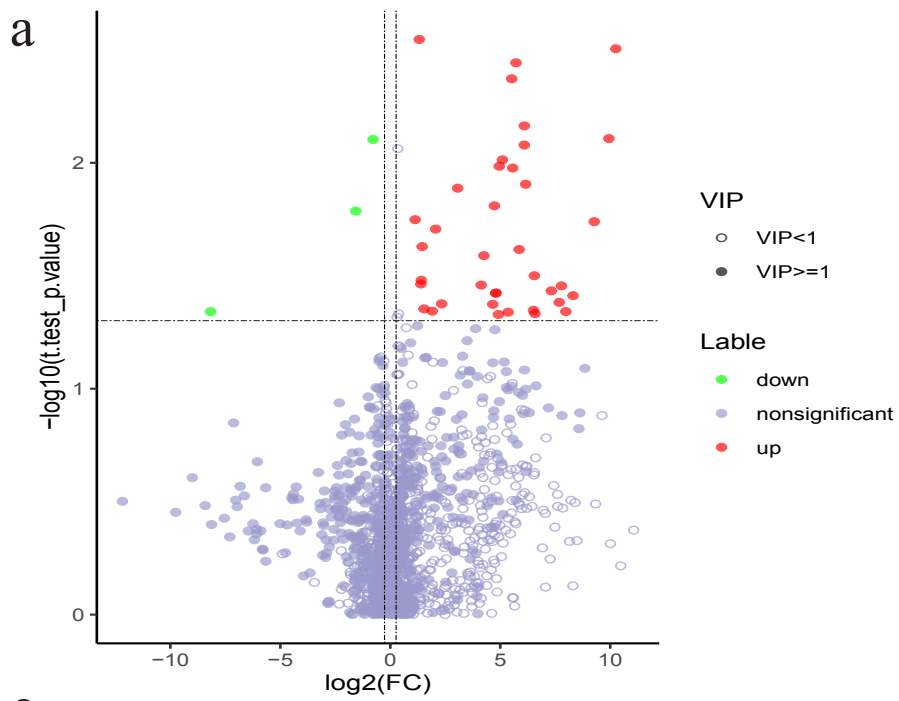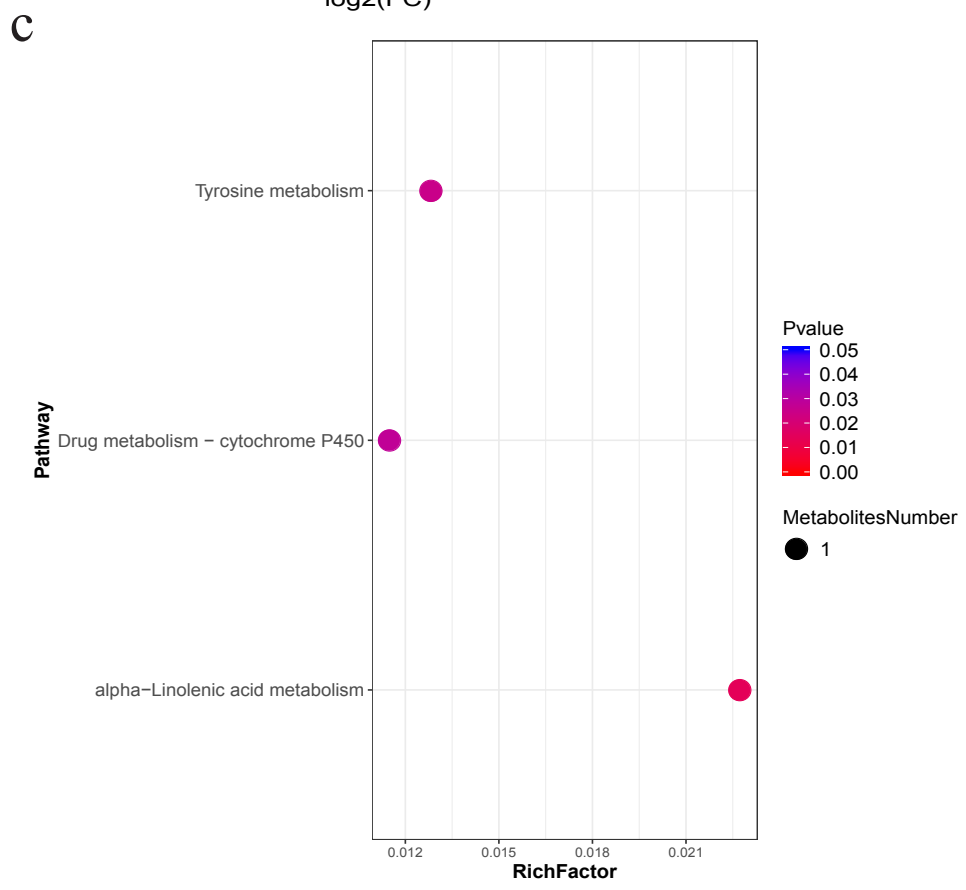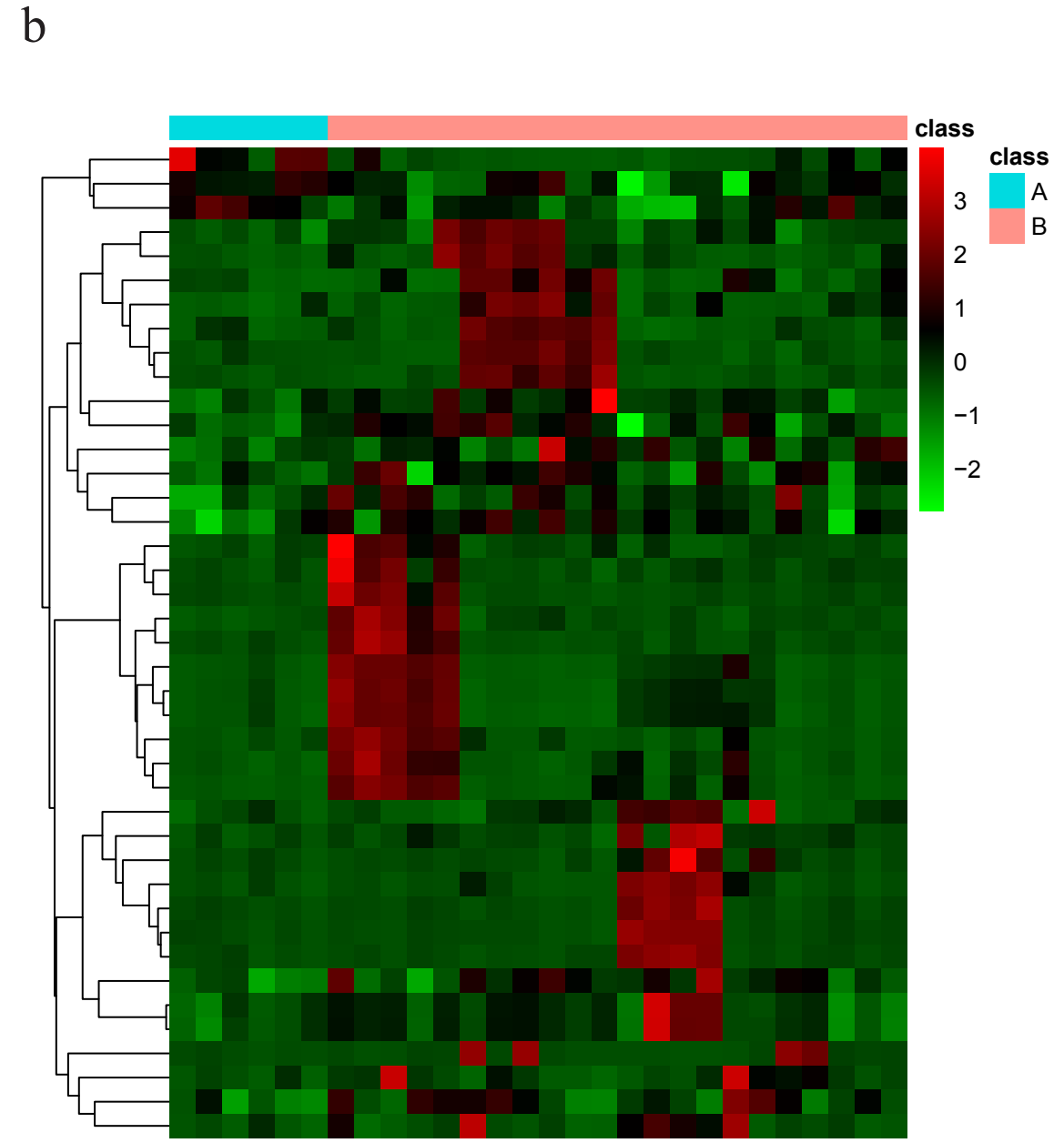

Supplement: Supplementary file 2 — Supplementary Information 2. [file 41598_2021_94256_MOESM2_ESM.pdf]

a

class —●— C —●— D

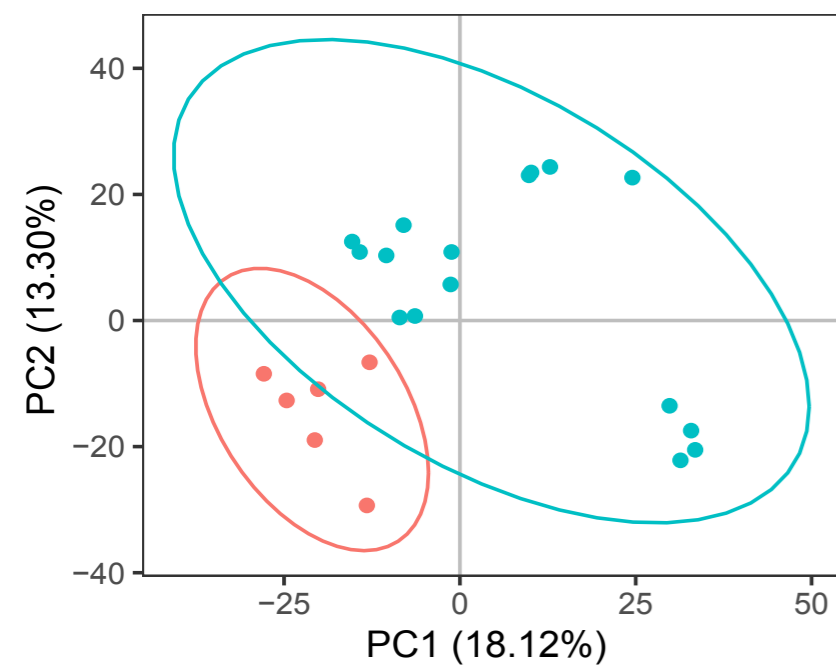

b

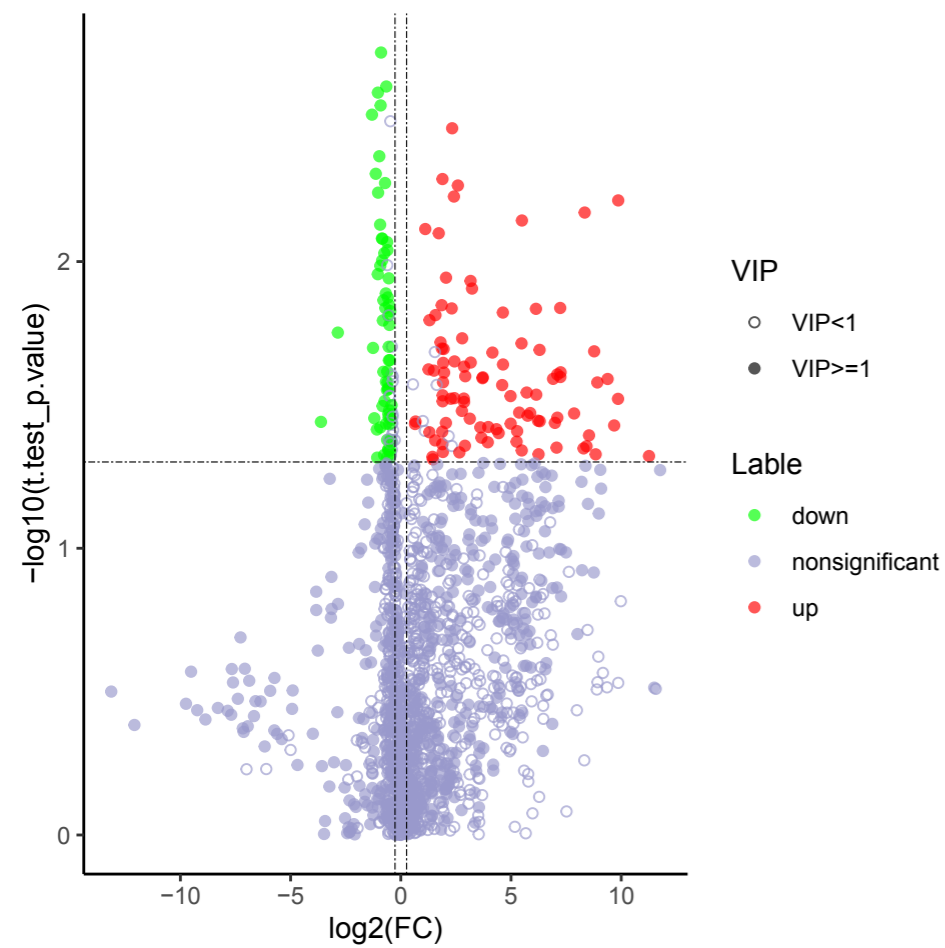

c

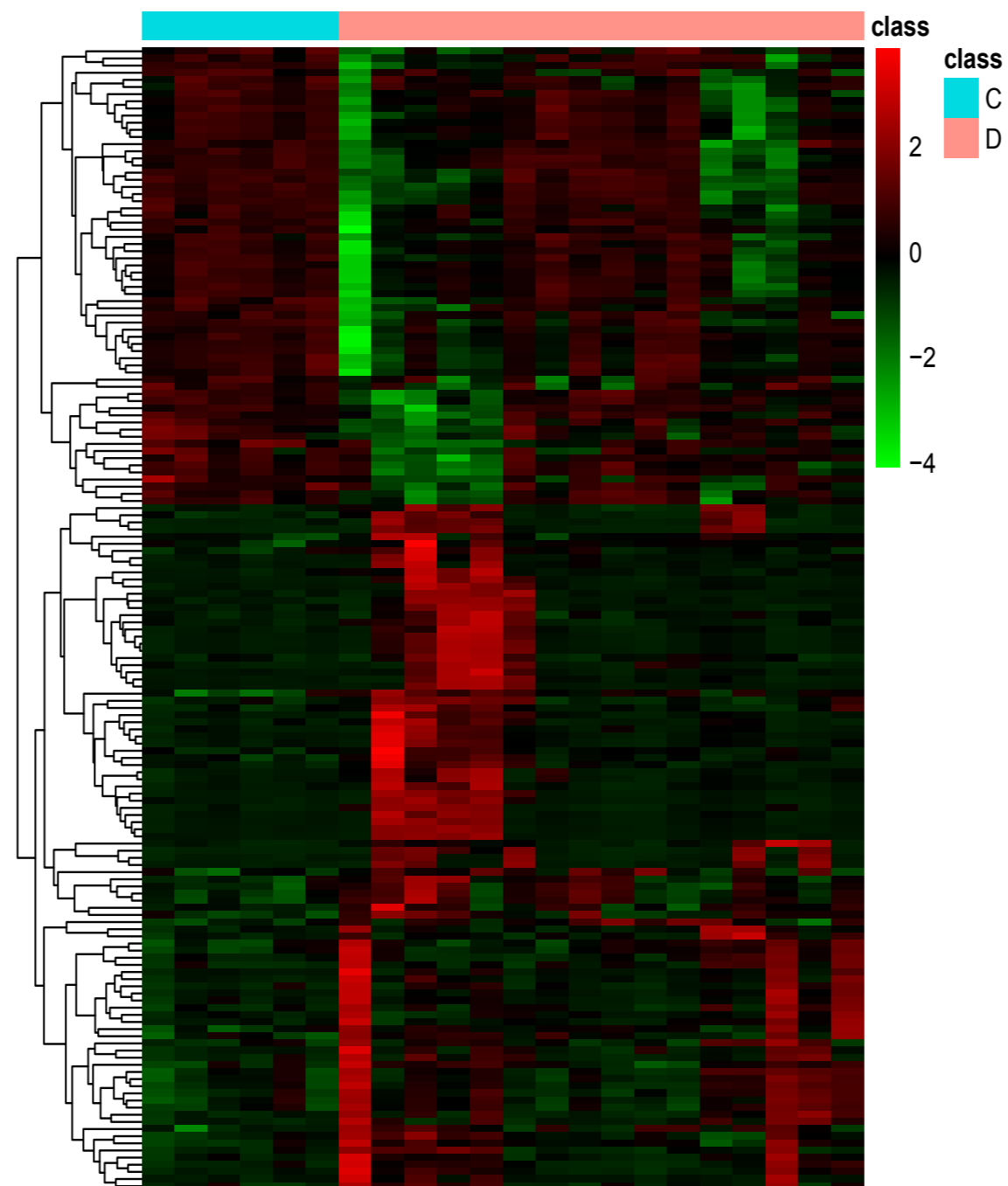

d

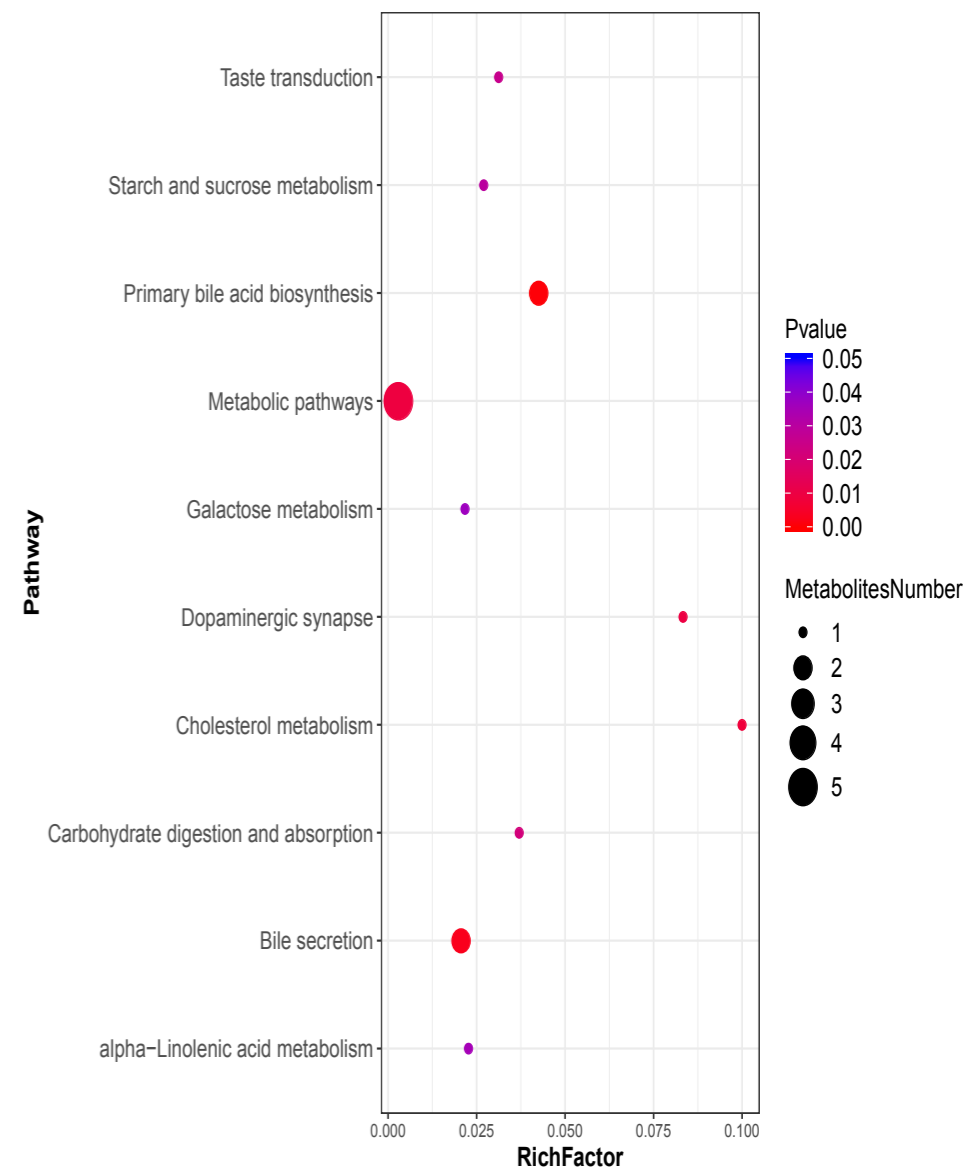

Supplement: Supplementary file 3 — Supplementary Information 3. [file 41598_2021_94256_MOESM3_ESM.pdf]
