# Supplementary material for: Proteomics and metabonomics analyses of Covid-19 complications in patients with pulmonary fibrosis
Source: Sci Rep. 2021 Jul 16;11:14601. doi: 10.1038/s41598-021-94256-8 (PMC8285535; doi:10.1038/s41598-021-94256-8)
Supplement: Supplementary file 4 — Supplementary Information 4. [file 41598_2021_94256_MOESM4_ESM.docx]

Proteomics and metabonomics analyses of Covid-19 complications in patients with pulmonary fibrosis

Jianrong Yang ^a, #^, Chunxia Chen ^b,c,#^, Wan Chen ^d, #^, Luying Huang ^e^ , Zhao Fu ^f^, Kun Ye ^g^, Liwen Lv ^d^, Zhihuang Nong ^h^, Xing Zhou ^i^, Wensheng Lu ^i, *^, Mei Zhong ^i, *^

^a^ Hepatobiliary Surgery, the People’s Hospital of Guangxi Zhuang Autonomous Region, Nanning, Guangxi 530021, P. R. China

^b^ Department of Research Center of Medical Sciences, the People’s Hospital of Guangxi Zhuang Autonomous Region, Nanning, Guangxi 530021, P. R. China

^c^ Department of Pharmaceutical Sciences (Shenzhen), Sun Yat-Sen University, Guangzhou, Guangdong 510006, P. R. China

^d^ Department of Emergency, the People’s Hospital of Guangxi Zhuang Autonomous Region, Nanning, Guangxi 530021, P. R. China

^e^ Department of Respiratory Diseases, the People’s Hospital of Guangxi Zhuang Autonomous Region, Nanning, Guangxi 530021, P. R. China

^f^ Department of Radiology, the People’s Hospital of Guangxi Zhuang Autonomous Region, Nanning, Guangxi 530021, P. R. China

^g^ Department of Nephrology, the People’s Hospital of Guangxi Zhuang Autonomous Region, Nanning, Guangxi 530021, P. R. China

^h^ Department of Pharmacy, the People’s Hospital of Guangxi Zhuang Autonomous Region, Nanning, Guangxi 530021, P. R. China

^i^ Department of Endocrinology, the People’s Hospital of Guangxi Zhuang Autonomous Region, Nanning, Guangxi 530021, P. R. China

^#^ First co-author, they contribute equally to this work.

* Co-corresponding authors

Supplementary figure legends

Fig S1. Proteomic analysis of Covid-19 patients with pulmonary fibrosis. a, Sample correlation heatmap. The darker the color, the higher the correlation. b, c, Enrichment analysis of DEPs by GO function (b) and KEGG function (c). d, KEGG Pathways enriched in DEPs from Covid-19 complicated with pulmonary fibrosis patients. A, Covid-19 patients without pulmonary fibrosis (n = 6). B, Covid-19 patients with pulmonary fibrosis (n = 22). RichFactor is defined as the number of differential metabolites annotated to the pathway divided by all identified metabolites annotated to the pathway.

Fig S2. Negative differentially expressed metabolites were identified, clustered and enriched. a, Volcano plots of negative compounds. Red dots, significantly upregulated metabolites; green dots, significantly downregulated metabolites. b, Heatmap of negative differentially expressed metabolites (“pheatmap” package in R software v3.5.0, https://www.r-project.org/). Each row denotes a different metabolite, and each column denotes a sample. c, Bubble plot of KEGG enrichment of negative metabolic pathways. The size of the circle dot denotes the number of different metabolites. A, Covid-19 patients without pulmonary fibrosis (n = 6). B, Covid-19 patients with pulmonary fibrosis (n = 22). RichFactor is defined as the number of differential metabolites annotated to the pathway divided by all identified metabolites annotated to the pathway.

Fig S3. Negative differentially expressed metabolites were identified and analyzed using proteomics in Covid-19 patients with progressive pulmonary fibrosis. a, PLS-DA model plot for negative ion mode. b, Volcano plots of negative compounds. c, Heatmap of different negative metabolites (“pheatmap” package in R software v3.5.0, https://www.r-project.org/). d, Bubble plot of KEGG enrichment of negative metabolic pathways. C, Nonprogressive pulmonary fibrosis of Covid-19 patients. D, Progressive pulmonary fibrosis of Covid-19 patients. RichFactor is defined as the number of differential metabolites annotated to the pathway divided by all identified metabolites annotated to the pathway.
