# Supplementary material for: Proteomics and metabonomics analyses of Covid-19 complications in patients with pulmonary fibrosis
Source: Sci Rep. 2021 Jul 16;11:14601. doi: 10.1038/s41598-021-94256-8 (PMC8285535; doi:10.1038/s41598-021-94256-8)
Supplement: Supplementary file 5 — Supplementary Information 5. [file 41598_2021_94256_MOESM5_ESM.docx]

Supplementary table 1. Comorbidity and prognosis of patients with Covid-19

| Index | All patients (n=85) | Nonpulmonary fibrosis (n=41) | Pulmonary fibrosis (n=44) |
| --- | --- | --- | --- |
| Comorbidity |  |  |  |
| Hypertension | 7 (8.2) | 1 (2.4) | 6 (13.6) |
| Diabetes | 4 (4.7) | 1 (2.4) | 3 (6.8) |
| Thyroid dysfunction | 3 (3.5) | 1 (2.4) | 2 (4.5) |
| Gastritis | 1 (1.2) | 0 (0) | 1 (2.3) |
| Bronchitis | 1 (1.2) | 0 (0) | 1 (2.3) |
| Tumor | 1 (1.2) | 1 (2.4) | 0 (0) |
| Renal artery embolism | 1 (1.2) | 0 (0) | 1 (2.3) |
| Cerebral infarction | 1 (1.2) | 0 (0) | 1 (2.3) |
| Prognosis |  |  |  |
| Survival | 84 (98.8) | 41 (100) | 43 (97.7) |
| Death | 1 (1.2) | 0 (0) | 1 (2.3) |

Note: data are cases of patients, with percentages in parentheses.
