# Supplementary material for: Proteomics and metabonomics analyses of Covid-19 complications in patients with pulmonary fibrosis
Source: Sci Rep. 2021 Jul 16;11:14601. doi: 10.1038/s41598-021-94256-8 (PMC8285535; doi:10.1038/s41598-021-94256-8)
Supplement: Supplementary file 6 — Supplementary Information 6. [file 41598_2021_94256_MOESM6_ESM.docx]

Supplementary table 2. A list of raw data from metabonomics and proteomics analyses and information on grouping numbers in articles

| Patient No. | Sample No. in original data | Grouping of Covid-19 patients with or without pulmonary fibrosis | Grouping of Covid-19 patients with or without progressive pulmonary fibrosis | Sample No. in proteomic analysis |
| --- | --- | --- | --- | --- |
| 1 | 20P03760003 | A | - | A_3 |
| 2 | 20P03760009 | A | - | A_9 |
| 3 | 20P03760010 | A | - | A_10 |
| 4 | 20P03760011 | A | - | A_11 |
| 5 | 20P03760018 | A | - | A_18 |
| 6 | 20P03760061 | A | - | A_23 |
| 7 | 20P03760001 | B | D | B_1 |
| 8 | 20P03760002 | B | C | B_2 |
| 9 | 20P03760004 | B | D | B_4 |
| 10 | 20P03760005 | B | D | B_5 |
| 11 | 20P03760006 | B | C | B_6 |
| 12 | 20P03760007 | B | D | B_7 |
| 13 | 20P03760008 | B | D | B_8 |
| 14 | 20P03760012 | B | D | B_12 |
| 15 | 20P03760013 | B | C | B_13 |
| 16 | 20P03760014 | B | C | B_14 |
| 17 | 20P03760015 | B | D | B_15 |
| 18 | 20P03760016 | B | D | B_16 |
| 19 | 20P03760017 | B | D | B_17 |
| 20 | 20P03760057 | B | D | B_19 |
| 21 | 20P03760058 | B | D | B_20 |
| 22 | 20P03760059 | B | D | B_21 |
| 23 | 20P03760060 | B | D | B_22 |
| 24 | 20P03760062 | B | D | B_24 |
| 25 | 20P03760063 | B | D | B_25 |
| 26 | 20P03760065 | B | C | B_26 |
| 27 | 20P03760066 | B | D | B_27 |
| 28 | 20P03760064 | B | C | - |

Note: A, Covid-19 patients without pulmonary fibrosis. B, Covid-19 patients with pulmonary fibrosis. C, Nonprogressive pulmonary fibrosis of Covid-19 patients. D, Progressive pulmonary fibrosis of Covid-19 patients. Since the protein content detected in patient 28 was very low, it was excluded from subsequent proteomic analysis.
