# Supplementary material for: Proteomics and metabonomics analyses of Covid-19 complications in patients with pulmonary fibrosis
Source: Sci Rep. 2021 Jul 16;11:14601. doi: 10.1038/s41598-021-94256-8 (PMC8285535; doi:10.1038/s41598-021-94256-8)
Supplement: Supplementary file 8 — Supplementary Information 8. [file 41598_2021_94256_MOESM8_ESM.docx]

Supplementary table 4. Differentially expressed proteins (DEPs) corresponding to enrichment pathways in proteomic analysis

| **Groups /No.** | **Pathway** | **Proteins**  **Database**\|ID\|Protein name |
| --- | --- | --- |
| **B vs. A** |  |  |
| 1 | Ribosome | sp\|P39019\|RS19_HUMAN  sp\|Q07020\|RL18_HUMAN |
| 2 | Glycosaminoglycan degradation | sp\|Q9Y251\|HPSE_HUMAN |
|  |  |  |
| **D vs. C** |  |  |
| 1 | Staphylococcus aureus infection | sp\|P09871\|C1S_HUMAN  tr\|A0A193CHQ9\|A0A193CHQ9_HUMAN  tr\|A0A1L2BU51\|A0A1L2BU51_HUMAN  tr\|A0A2U8J8X1\|A0A2U8J8X1_HUMAN  tr\|A0A3B3ISW6\|A0A3B3ISW6_HUMAN tr\|A0A449C1A8\|A0A449C1A8_HUMAN tr\|A0N7I9\|A0N7I9_HUMAN  tr\|A2J1M2\|A2J1M2_HUMAN tr\|A2J1N7\|A2J1N7_HUMAN tr\|A2N2G5\|A2N2G5_HUMAN tr\|A2NXP8\|A2NXP8_HUMAN tr\|Q7Z351\|Q7Z351_HUMAN tr\|S6B2A6\|S6B2A6_HUMAN |
| 2 | Primary immunodeficiency | tr\|A0A193CHQ9\|A0A193CHQ9_HUMAN tr\|A0A1L2BU51\|A0A1L2BU51_HUMAN tr\|A0A2U8J8X1\|A0A2U8J8X1_HUMAN tr\|A0A449C1A8\|A0A449C1A8_HUMAN tr\|A0N7I9\|A0N7I9_HUMAN tr\|A2J1M2\|A2J1M2_HUMAN tr\|A2J1N7\|A2J1N7_HUMAN tr\|A2N2G5\|A2N2G5_HUMAN tr\|A2NXP8\|A2NXP8_HUMAN tr\|Q7Z351\|Q7Z351_HUMAN  tr\|S6B2A6\|S6B2A6_HUMAN |
| 3 | Asthma | tr\|A0A193CHQ9\|A0A193CHQ9_HUMAN tr\|A0A1L2BU51\|A0A1L2BU51_HUMAN tr\|A0A2U8J8X1\|A0A2U8J8X1_HUMAN  tr\|A0A449C1A8\|A0A449C1A8_HUMAN tr\|A0N7I9\|A0N7I9_HUMAN tr\|A2J1M2\|A2J1M2_HUMAN tr\|A2J1N7\|A2J1N7_HUMAN tr\|A2N2G5\|A2N2G5_HUMAN tr\|A2NXP8\|A2NXP8_HUMAN tr\|Q7Z351\|Q7Z351_HUMAN tr\|S6B2A6\|S6B2A6_HUMAN |
| 4 | Measles | tr\|A0A193CHQ9\|A0A193CHQ9_HUMAN tr\|A0A1L2BU51\|A0A1L2BU51_HUMAN tr\|A0A2U8J8X1\|A0A2U8J8X1_HUMAN tr\|A0A449C1A8\|A0A449C1A8_HUMAN tr\|A0N7I9\|A0N7I9_HUMAN  tr\|A2J1M2\|A2J1M2_HUMAN  tr\|A2J1N7\|A2J1N7_HUMAN tr\|A2N2G5\|A2N2G5_HUMAN tr\|A2NXP8\|A2NXP8_HUMAN tr\|Q7Z351\|Q7Z351_HUMAN tr\|S6B2A6\|S6B2A6_HUMAN |
| 5 | Leishmaniasis | tr\|A0A193CHQ9\|A0A193CHQ9_HUMAN tr\|A0A1L2BU51\|A0A1L2BU51_HUMAN tr\|A0A2U8J8X1\|A0A2U8J8X1_HUMAN tr\|A0A449C1A8\|A0A449C1A8_HUMAN  tr\|A0N7I9\|A0N7I9_HUMAN  tr\|A2J1M2\|A2J1M2_HUMAN tr\|A2J1N7\|A2J1N7_HUMAN tr\|A2N2G5\|A2N2G5_HUMAN  tr\|A2NXP8\|A2NXP8_HUMAN tr\|Q7Z351\|Q7Z351_HUMAN tr\|S6B2A6\|S6B2A6_HUMAN |
| 6 | Intestinal immune network for IgA production | tr\|A0A193CHQ9\|A0A193CHQ9_HUMAN tr\|A0A1L2BU51\|A0A1L2BU51_HUMAN tr\|A0A2U8J8X1\|A0A2U8J8X1_HUMAN tr\|A0A449C1A8\|A0A449C1A8_HUMAN tr\|A0N7I9\|A0N7I9_HUMAN  tr\|A2J1M2\|A2J1M2_HUMAN  tr\|A2J1N7\|A2J1N7_HUMAN tr\|A2N2G5\|A2N2G5_HUMAN tr\|A2NXP8\|A2NXP8_HUMAN tr\|Q7Z351\|Q7Z351_HUMAN tr\|S6B2A6\|S6B2A6_HUMAN |
| 7 | Rheumatoid arthritis | tr\|A0A193CHQ9\|A0A193CHQ9_HUMAN tr\|A0A1L2BU51\|A0A1L2BU51_HUMAN tr\|A0A2U8J8X1\|A0A2U8J8X1_HUMAN tr\|A0A449C1A8\|A0A449C1A8_HUMAN tr\|A0N7I9\|A0N7I9_HUMAN tr\|A2J1M2\|A2J1M2_HUMAN tr\|A2J1N7\|A2J1N7_HUMAN  tr\|A2N2G5\|A2N2G5_HUMAN tr\|A2NXP8\|A2NXP8_HUMAN tr\|Q7Z351\|Q7Z351_HUMAN  tr\|S6B2A6\|S6B2A6_HUMAN |
| 8 | Fc epsilon RI signaling pathway | tr\|A0A193CHQ9\|A0A193CHQ9_HUMAN  tr\|A0A1L2BU51\|A0A1L2BU51_HUMAN tr\|A0A2U8J8X1\|A0A2U8J8X1_HUMAN  tr\|A0A449C1A8\|A0A449C1A8_HUMAN  tr\|A0N7I9\|A0N7I9_HUMAN  tr\|A2J1M2\|A2J1M2_HUMAN  tr\|A2J1N7\|A2J1N7_HUMAN tr\|A2N2G5\|A2N2G5_HUMAN  tr\|A2NXP8\|A2NXP8_HUMAN  tr\|Q7Z351\|Q7Z351_HUMAN  tr\|S6B2A6\|S6B2A6_HUMAN |
| 9 | Phospholipase D signaling pathway | tr\|A0A193CHQ9\|A0A193CHQ9_HUMAN  tr\|A0A1L2BU51\|A0A1L2BU51_HUMAN  tr\|A0A2U8J8X1\|A0A2U8J8X1_HUMAN  tr\|A0A449C1A8\|A0A449C1A8_HUMAN  tr\|A0N7I9\|A0N7I9_HUMAN  tr\|A2J1M2\|A2J1M2_HUMAN  tr\|A2J1N7\|A2J1N7_HUMAN  tr\|A2N2G5\|A2N2G5_HUMAN  tr\|A2NXP8\|A2NXP8_HUMAN  tr\|Q7Z351\|Q7Z351_HUMAN  tr\|S6B2A6\|S6B2A6_HUMAN |
| 10 | Hematopoietic cell lineage | tr\|A0A193CHQ9\|A0A193CHQ9_HUMAN  tr\|A0A1L2BU51\|A0A1L2BU51_HUMAN  tr\|A0A2U8J8X1\|A0A2U8J8X1_HUMAN  tr\|A0A449C1A8\|A0A449C1A8_HUMAN  tr\|A0N7I9\|A0N7I9_HUMAN  tr\|A2J1M2\|A2J1M2_HUMAN  tr\|A2J1N7\|A2J1N7_HUMAN  tr\|A2N2G5\|A2N2G5_HUMAN  tr\|A2NXP8\|A2NXP8_HUMAN  tr\|Q7Z351\|Q7Z351_HUMAN  tr\|S6B2A6\|S6B2A6_HUMAN |
| 11 | Calcium signaling pathway | tr\|A0A193CHQ9\|A0A193CHQ9_HUMAN tr\|A0A1L2BU51\|A0A1L2BU51_HUMAN tr\|A0A2U8J8X1\|A0A2U8J8X1_HUMAN  tr\|A0A449C1A8\|A0A449C1A8_HUMAN  tr\|A0N7I9\|A0N7I9_HUMAN  tr\|A2J1M2\|A2J1M2_HUMAN  tr\|A2J1N7\|A2J1N7_HUMAN  tr\|A2N2G5\|A2N2G5_HUMAN  tr\|A2NXP8\|A2NXP8_HUMAN  tr\|Q7Z351\|Q7Z351_HUMAN  tr\|S6B2A6\|S6B2A6_HUMAN |
| 12 | B cell receptor signaling pathway | tr\|A0A193CHQ9\|A0A193CHQ9_HUMAN tr\|A0A1L2BU51\|A0A1L2BU51_HUMAN  tr\|A0A2U8J8X1\|A0A2U8J8X1_HUMAN tr\|A0A449C1A8\|A0A449C1A8_HUMAN  tr\|A0N7I9\|A0N7I9_HUMAN  tr\|A2J1M2\|A2J1M2_HUMAN  tr\|A2J1N7\|A2J1N7_HUMAN  tr\|A2N2G5\|A2N2G5_HUMAN  tr\|A2NXP8\|A2NXP8_HUMAN  tr\|Q7Z351\|Q7Z351_HUMAN  tr\|S6B2A6\|S6B2A6_HUMAN |
| 13 | Autoimmune thyroid disease | tr\|A0A193CHQ9\|A0A193CHQ9_HUMAN  tr\|A0A1L2BU51\|A0A1L2BU51_HUMAN  tr\|A0A2U8J8X1\|A0A2U8J8X1_HUMAN  tr\|A0A449C1A8\|A0A449C1A8_HUMAN  tr\|A0N7I9\|A0N7I9_HUMAN  tr\|A2J1M2\|A2J1M2_HUMAN  tr\|A2J1N7\|A2J1N7_HUMAN  tr\|A2N2G5\|A2N2G5_HUMAN  tr\|A2NXP8\|A2NXP8_HUMAN  tr\|Q7Z351\|Q7Z351_HUMAN  tr\|S6B2A6\|S6B2A6_HUMAN |
| 14 | Allograft rejection | tr\|A0A193CHQ9\|A0A193CHQ9_HUMAN  tr\|A0A1L2BU51\|A0A1L2BU51_HUMAN  tr\|A0A2U8J8X1\|A0A2U8J8X1_HUMAN  tr\|A0A449C1A8\|A0A449C1A8_HUMAN  tr\|A0N7I9\|A0N7I9_HUMAN  tr\|A2J1M2\|A2J1M2_HUMAN  tr\|A2J1N7\|A2J1N7_HUMAN  tr\|A2N2G5\|A2N2G5_HUMAN  tr\|A2NXP8\|A2NXP8_HUMAN  tr\|Q7Z351\|Q7Z351_HUMAN  tr\|S6B2A6\|S6B2A6_HUMAN |
| 15 | Natural killer cell mediated cytotoxicity | tr\|A0A193CHQ9\|A0A193CHQ9_HUMAN  tr\|A0A1L2BU51\|A0A1L2BU51_HUMAN  tr\|A0A2U8J8X1\|A0A2U8J8X1_HUMAN  tr\|A0A449C1A8\|A0A449C1A8_HUMAN  tr\|A0N7I9\|A0N7I9_HUMAN  tr\|A2J1M2\|A2J1M2_HUMAN  tr\|A2J1N7\|A2J1N7_HUMAN  tr\|A2N2G5\|A2N2G5_HUMAN  tr\|A2NXP8\|A2NXP8_HUMAN  tr\|Q7Z351\|Q7Z351_HUMAN  tr\|S6B2A6\|S6B2A6_HUMAN |
| 16 | Carbon metabolism | sp\|P04040\|CATA_HUMAN sp\|P05062\|ALDOB_HUMAN |
| 17 | Systemic lupus erythematosus | sp\|P09871\|C1S_HUMAN  tr\|A0A193CHQ9\|A0A193CHQ9_HUMAN  tr\|A0A1L2BU51\|A0A1L2BU51_HUMAN  tr\|A0A2U8J8X1\|A0A2U8J8X1_HUMAN  tr\|A0A449C1A8\|A0A449C1A8_HUMAN  tr\|A0N7I9\|A0N7I9_HUMAN  tr\|A2J1M2\|A2J1M2_HUMAN  tr\|A2J1N7\|A2J1N7_HUMAN  tr\|A2N2G5\|A2N2G5_HUMAN  tr\|A2NXP8\|A2NXP8_HUMAN  tr\|Q7Z351\|Q7Z351_HUMAN  tr\|S6B2A6\|S6B2A6_HUMAN |
| 18 | NF-kappa B signaling pathway | tr\|A0A193CHQ9\|A0A193CHQ9_HUMAN tr\|A0A1L2BU51\|A0A1L2BU51_HUMAN tr\|A0A2U8J8X1\|A0A2U8J8X1_HUMAN tr\|A0A449C1A8\|A0A449C1A8_HUMAN  tr\|A0N7I9\|A0N7I9_HUMAN  tr\|A2J1M2\|A2J1M2_HUMAN  tr\|A2J1N7\|A2J1N7_HUMAN  tr\|A2N2G5\|A2N2G5_HUMAN  tr\|A2NXP8\|A2NXP8_HUMAN  tr\|Q7Z351\|Q7Z351_HUMAN  tr\|S6B2A6\|S6B2A6_HUMAN |
| 19 | Fc gamma R-mediated phagocytosis | tr\|A0A193CHQ9\|A0A193CHQ9_HUMAN  tr\|A0A1L2BU51\|A0A1L2BU51_HUMAN  tr\|A0A2U8J8X1\|A0A2U8J8X1_HUMAN  tr\|A0A449C1A8\|A0A449C1A8_HUMAN  tr\|A0N7I9\|A0N7I9_HUMAN  tr\|A2J1M2\|A2J1M2_HUMAN  tr\|A2J1N7\|A2J1N7_HUMAN  tr\|A2N2G5\|A2N2G5_HUMAN  tr\|A2NXP8\|A2NXP8_HUMAN  tr\|Q7Z351\|Q7Z351_HUMAN  tr\|S6B2A6\|S6B2A6_HUMAN |
| 20 | Dilated cardiomyopathy (DCM) | tr\|A0A193CHQ9\|A0A193CHQ9_HUMAN tr\|A0A1L2BU51\|A0A1L2BU51_HUMAN  tr\|A0A2U8J8X1\|A0A2U8J8X1_HUMAN  tr\|A0A449C1A8\|A0A449C1A8_HUMAN  tr\|A0N7I9\|A0N7I9_HUMAN  tr\|A2J1M2\|A2J1M2_HUMAN  tr\|A2J1N7\|A2J1N7_HUMAN  tr\|A2N2G5\|A2N2G5_HUMAN  tr\|A2NXP8\|A2NXP8_HUMAN  tr\|Q7Z351\|Q7Z351_HUMAN  tr\|S6B2A6\|S6B2A6_HUMAN |
| 21 | Tuberculosis | tr\|A0A193CHQ9\|A0A193CHQ9_HUMAN  tr\|A0A1L2BU51\|A0A1L2BU51_HUMAN tr\|A0A2U8J8X1\|A0A2U8J8X1_HUMAN tr\|A0A449C1A8\|A0A449C1A8_HUMAN tr\|A0N7I9\|A0N7I9_HUMAN  tr\|A2J1M2\|A2J1M2_HUMAN  tr\|A2J1N7\|A2J1N7_HUMAN  tr\|A2N2G5\|A2N2G5_HUMAN  tr\|A2NXP8\|A2NXP8_HUMAN  tr\|Q7Z351\|Q7Z351_HUMAN  tr\|S6B2A6\|S6B2A6_HUMAN |
| 22 | Viral myocarditis | tr\|A0A193CHQ9\|A0A193CHQ9_HUMAN  tr\|A0A1L2BU51\|A0A1L2BU51_HUMAN  tr\|A0A2U8J8X1\|A0A2U8J8X1_HUMAN  tr\|A0A449C1A8\|A0A449C1A8_HUMAN  tr\|A0N7I9\|A0N7I9_HUMAN  tr\|A2J1M2\|A2J1M2_HUMAN  tr\|A2J1N7\|A2J1N7_HUMAN  tr\|A2N2G5\|A2N2G5_HUMAN  tr\|A2NXP8\|A2NXP8_HUMAN  tr\|Q7Z351\|Q7Z351_HUMAN  tr\|S6B2A6\|S6B2A6_HUMAN |
| 23 | Epstein-Barr virus infection | tr\|A0A193CHQ9\|A0A193CHQ9_HUMAN  tr\|A0A1L2BU51\|A0A1L2BU51_HUMAN  tr\|A0A2U8J8X1\|A0A2U8J8X1_HUMAN  tr\|A0A449C1A8\|A0A449C1A8_HUMAN  tr\|A0N7I9\|A0N7I9_HUMAN  tr\|A2J1M2\|A2J1M2_HUMAN  tr\|A2J1N7\|A2J1N7_HUMAN  tr\|A2N2G5\|A2N2G5_HUMAN  tr\|A2NXP8\|A2NXP8_HUMAN  tr\|Q7Z351\|Q7Z351_HUMAN  tr\|S6B2A6\|S6B2A6_HUMAN |
| 24 | Amoebiasis | tr\|A0A193CHQ9\|A0A193CHQ9_HUMAN  tr\|A0A1L2BU51\|A0A1L2BU51_HUMAN  tr\|A0A2U8J8X1\|A0A2U8J8X1_HUMAN  tr\|A0A449C1A8\|A0A449C1A8_HUMAN  tr\|A0N7I9\|A0N7I9_HUMAN  tr\|A2J1M2\|A2J1M2_HUMAN  tr\|A2J1N7\|A2J1N7_HUMAN  tr\|A2N2G5\|A2N2G5_HUMAN  tr\|A2NXP8\|A2NXP8_HUMAN  tr\|Q7Z351\|Q7Z351_HUMAN  tr\|S6B2A6\|S6B2A6_HUMAN |
| 25 | Transcriptional misregulation in cancer | tr\|A0A193CHQ9\|A0A193CHQ9_HUMAN  tr\|A0A1L2BU51\|A0A1L2BU51_HUMAN  tr\|A0A2U8J8X1\|A0A2U8J8X1_HUMAN  tr\|A0A449C1A8\|A0A449C1A8_HUMAN  tr\|A0N7I9\|A0N7I9_HUMAN  tr\|A2J1M2\|A2J1M2_HUMAN  tr\|A2J1N7\|A2J1N7_HUMAN  tr\|A2N2G5\|A2N2G5_HUMAN  tr\|A2NXP8\|A2NXP8_HUMAN  tr\|Q7Z351\|Q7Z351_HUMAN  tr\|S6B2A6\|S6B2A6_HUMAN |
| 26 | African trypanosomiasis | tr\|A0A193CHQ9\|A0A193CHQ9_HUMAN  tr\|A0A1L2BU51\|A0A1L2BU51_HUMAN  tr\|A0A2U8J8X1\|A0A2U8J8X1_HUMAN  tr\|A0A449C1A8\|A0A449C1A8_HUMAN  tr\|A0N7I9\|A0N7I9_HUMAN  tr\|A2J1M2\|A2J1M2_HUMAN  tr\|A2J1N7\|A2J1N7_HUMAN  tr\|A2N2G5\|A2N2G5_HUMAN  tr\|A2NXP8\|A2NXP8_HUMAN  tr\|Q7Z351\|Q7Z351_HUMAN  tr\|S6B2A6\|S6B2A6_HUMAN |
| 27 | Tryptophan metabolism | sp\|P04040\|CATA_HUMAN |
| 28 | Glyoxylate and dicarboxylate metabolism | sp\|P04040\|CATA_HUMAN |
| 29 | Phagosome | tr\|A0A193CHQ9\|A0A193CHQ9_HUMAN  tr\|A0A1L2BU51\|A0A1L2BU51_HUMAN  tr\|A0A2U8J8X1\|A0A2U8J8X1_HUMAN  tr\|A0A449C1A8\|A0A449C1A8_HUMAN  tr\|A0N7I9\|A0N7I9_HUMAN  tr\|A2J1M2\|A2J1M2_HUMAN  tr\|A2J1N7\|A2J1N7_HUMAN  tr\|A2N2G5\|A2N2G5_HUMAN  tr\|A2NXP8\|A2NXP8_HUMAN  tr\|Q7Z351\|Q7Z351_HUMAN  tr\|S6B2A6\|S6B2A6_HUMAN |
| 30 | PI3K-Akt signaling pathway | tr\|A0A193CHQ9\|A0A193CHQ9_HUMAN  tr\|A0A1L2BU51\|A0A1L2BU51_HUMAN  tr\|A0A2U8J8X1\|A0A2U8J8X1_HUMAN  tr\|A0A449C1A8\|A0A449C1A8_HUMAN  tr\|A0N7I9\|A0N7I9_HUMAN  tr\|A2J1M2\|A2J1M2_HUMAN  tr\|A2J1N7\|A2J1N7_HUMAN  tr\|A2N2G5\|A2N2G5_HUMAN  tr\|A2NXP8\|A2NXP8_HUMAN  tr\|Q7Z351\|Q7Z351_HUMAN  tr\|S6B2A6\|S6B2A6_HUMAN |

Note: A, Covid-19 patients without pulmonary fibrosis. B, Covid-19 patients with pulmonary fibrosis. C, Nonprogressive pulmonary fibrosis of Covid-19 patients. D, Progressive pulmonary fibrosis of Covid-19 patients. sp, Swiss-Prot database. tr, TrEMBL database.
