# Supplementary material for: Proteomics and metabonomics analyses of Covid-19 complications in patients with pulmonary fibrosis
Source: Sci Rep. 2021 Jul 16;11:14601. doi: 10.1038/s41598-021-94256-8 (PMC8285535; doi:10.1038/s41598-021-94256-8)
Supplement: Supplementary file 11 — Supplementary Information 11. [file 41598_2021_94256_MOESM11_ESM.docx]

Supplementary table 7. Differentially expressed metabolites corresponding to enrichment pathways in metabolomic analysis

| **Groups /No.** | **Pathway** | **Metbolites** |
| --- | --- | --- |
| **B vs. A,** Negative metabolites |  |  |
| 1 | alpha-Linolenic acid metabolism | Traumatic acid |
| 2 | Tyrosine metabolism | 3,4-dihydroxyphenyl glycol |
| 3 | Drug metabolism - cytochrome P450 | 2-hydroxyfelbamate |
|  |  |  |
| **B vs. A,** Positive metabolites |  |  |
| 1 | PPAR signaling pathway | Leukotriene b4 |
| 2 | D-Arginine and D-ornithine metabolism | D-ornithine |
| 3 | Insulin resistance | O-acetylcarnitine |
| 4 | Caffeine metabolism | Caffeine |
| 5 | Inflammatory mediator regulation of TRP channels | Leukotriene b4 |
| 6 | Serotonergic synapse | Leukotriene b4 |
|  |  |  |
| **D vs. C** Negative metabolites |  |  |
| 1 | Primary bile acid biosynthesis | Glycocholic acid  Cholic acid |
| 2 | Bile secretion | Glycocholic acid  Cholic acid |
| 3 | Cholesterol metabolism | Glycocholic acid |
| 4 | Metabolic pathways | Sucrose  Homovanillic acid  Glycocholic acid  Cholic acid  Boldione |
| 5 | Dopaminergic synapse | Homovanillic acid |
| 6 | Carbohydrate digestion and absorption | Sucrose |
| 7 | Taste transduction | Sucrose |
| 8 | Starch and sucrose metabolism | Sucrose |
| 9 | alpha-Linolenic acid metabolism | Traumatic acid |
| 10 | Galactose metabolism | Sucrose |
|  |  |  |
| **D vs. C** Positive metabolites |  |  |
| 1 | Bile secretion | Spermidine  Spermine  Leukotriene b4  Glycocholate  Cholate  Taurocholic acid |
| 2 | Metabolic pathways | Spermidine  Spermine  L-arginine  Urocanate  Pyroglutamate  Hypoxanthine  N-acetylornithine  Indole-3-acetaldehyde  4-hydroxybutyric acid (ghb)  Mhpg  Cortisone  Leukotriene b4  Glycocholate  Cholate  Taurocholic acid  D-erythro-sphingosine 1-phosphate |
| 3 | Glutathione metabolism | Spermidine  Spermine  Pyroglutamate |
| 4 | Primary bile acid biosynthesis | Glycocholate  Cholate  Taurocholic acid |
| 5 | Cholesterol metabolism | Glycocholate  Taurocholic acid |
| 6 | Arginine and proline metabolism | Spermidine  Spermine  L-arginine |
| 7 | Arginine biosynthesis | L-arginine  N-acetylornithine |
| 8 | beta-Alanine metabolism | Spermidine  Spermine |
| 9 | Inflammatory mediator regulation of TRP channels | Leukotriene b4  Icomucret |
| 10 | Neuroactive ligand-receptor interaction | Leukotriene b4  D-erythro-sphingosine 1-phosphate |
| 11 | mTOR signaling pathway | L-arginine |
| 12 | Salmonella infection | L-arginine |
| 13 | PPAR signaling pathway | Leukotriene b4 |
| 14 | Arachidonic acid metabolism | Leukotriene b4  Icomucret |
| 15 | Chagas disease (American trypanosomiasis) | L-arginine |
| 16 | Tryptophan metabolism | Indole-3-acetaldehyde  N-methyltryptamine |
| 17 | Aldosterone-regulated sodium reabsorption | Cortisone |
| 18 | Fc gamma R-mediated phagocytosis | D-erythro-sphingosine 1-phosphate |
| 19 | Tuberculosis | D-erythro-sphingosine 1-phosphate |
| 20 | Apelin signaling pathway | D-erythro-sphingosine 1-phosphate |
| 21 | D-Arginine and D-ornithine metabolism | L-arginine |
| 22 | Amyotrophic lateral sclerosis (ALS) | L-arginine |
| 23 | Calcium signaling pathway | D-erythro-sphingosine 1-phosphate |
| 24 | Prostate cancer | Cortisone |
| 25 | Phospholipase D signaling pathway | D-erythro-sphingosine 1-phosphate |
| 26 | Amoebiasis | L-arginine |
| 27 | Sphingolipid signaling pathway | D-erythro-sphingosine 1-phosphate |
| 28 | ABC transporters | Spermidine  L-arginine |
| 29 | Biosynthesis of amino acids | L-arginine  N-acetylornithine |
| 30 | Taurine and hypotaurine metabolism | Taurocholic acid |

Note: A, Covid-19 patients without pulmonary fibrosis. B, Covid-19 patients with pulmonary fibrosis. C, Nonprogressive pulmonary fibrosis of Covid-19 patients. D, Progressive pulmonary fibrosis of Covid-19 patients.
